# SUMO E3 ligase MUL1 inhibits lymph node metastasis of bladder cancer by mediating mitochondrial HSPA9 translocation

**DOI:** 10.7150/ijbs.98772

**Published:** 2024-07-15

**Authors:** Jilin Wu, Ming Huang, Wen Dong, Yuelong Chen, Qianghua Zhou, Qiang Zhang, Junjiong Zheng, Yeqing Liu, Yangjie Zhang, Sen Liu, Chenwei Yang, Siting Chen, Jian Huang, Tianxin Lin, Xu Chen

**Affiliations:** 1 Department of Urology, Sun Yat-sen Memorial Hospital, Sun Yat-sen University, Guangzhou, Guangdong, 510120, China.; 2 Guangdong Provincial Key Laboratory of Malignant Tumor Epigenetics and Gene Regulation, Sun Yat-Sen Memorial Hospital, Sun Yat-Sen University, Guangzhou, Guangdong, 510120, China.; 3 Guangdong Provincial Clinical Research Center for Urological Diseases, Guangzhou, Guangdong, 510120, China.; 4 Department of Pathology, Sun Yat-sen Memorial Hospital, Sun Yat-sen University, Guangzhou, Guangdong, 510120, China.

**Keywords:** Bladder cancer, Lymph node metastasis, MUL1, HSPA9, PRC2.

## Abstract

Lymph node (LN) metastasis is the dominant cause of death in bladder cancer (BCa) patients, but the underlying mechanism remains largely unknown. In recent years, accumulating studies have confirmed that bidirectional mitochondria-nucleus communication is essential for sustaining multiple function of mitochondria. However, little has been studied regarding whether and how the translocation of mitochondrial proteins is involved in LN metastasis. In this study, we first identified that the SUMO E3 ligase MUL1 was significantly downregulated in LN-metastatic BCa tissues and correlated with a good prognosis. Mechanistically, MUL1 SUMOylated HSPA9 at the K612 residue, leading to HSPA9 export from mitochondria and interaction with SUZ12 and in the nucleus. Consequently, MUL1 induced the ubiquitination-mediated degradation of SUZ12 and EZH2 and induced downstream STAT3 pathway inhibition in a HSPA9-dependent manner. Importantly, mutation of HSPA9 SUMO-conjugation motifs limited the translocation of mitochondrial HSPA9 and blocked the HSPA9-SUZ12 and HSPA9-EZH2 interactions. With mutation of the HSPA9 K612 site, the suppressive role of MUL1 overexpression was lost in BCa cells. Further *in vitro* and *in vivo* assays revealed that MUL1 inhibits the metastasis and proliferation of BCa cells. Overall, our study reveals a novel function and molecular mechanism of SUMO E3 ligases in LN metastasis.

## Introduction

Bladder cancer (BCa) ranks as the second most prevalent malignancy affecting the urinary system globally, with a tendency toward lymph node (LN) metastasis 1. Significantly, LN metastasis status is strongly correlated with the prognosis of BCa patients 2, and the 5-year overall survival (OS) rate of BCa patients decreases to less than 30% once LN metastasis occurs 3, 4. Our previous studies verified that the evolution of metastatic and disseminated traits in BCa cells was a critical process in LN metastasis 5-7. However, the mechanisms underlying aberrant inactivation or hyperactivation of central cellular signaling pathways that convert cancer cells to highly invasive and metastatic phenotypes remain largely unknown.

It is well established that cancer cell motility and invasion take advantage of the ATP generated by mitochondria through oxidative phosphorylation 8, 9. Meanwhile, mitophagy 10, mitochondrial fission 11, and the mitochondrial unfolded protein response 12 have also been found to be involved in cancer metastasis. Intriguingly, 99% of mitochondrial proteins are translated from nuclear DNA and imported into mitochondria 13, while altered mitochondrial metabolic or genomic signals would cause epigenetic changes in nucleus 14, 15. These findings suggest that bidirectional mitochondria-nucleus communication is important in sustaining the multiple functions of mitochondria. In recent years, small ubiquitin-like modifier (SUMO) binding (SUMOylation) has been demonstrated to regulate the activation, stability and subcellular location of key molecules in cancer cells 16-19. Shangguan et al. demonstrated that the cytosolic fraction without mitochondria contains a higher expression of SUMOylated HK2 compared to the mitochondrial fraction. Conversely, de-SUMOylated HK2 binds to mitochondria and enhances both prostate cancer cell proliferation and resistance to chemotherapy.^20^. Additionally, SUMOylation of the fusion protein SYNJ2BP-COX16 induced the binding of SYNJ2BP-COX16 to mitochondria and promoted lung metastasis in breast cancer cells 21. Together, these studies revealed that SUMOylation catalyzes specific mitochondrial protein translocation to enhance cancer progression. However, the role of SUMOylation-mediated mitochondrial protein translocation in LN metastasis has never been investigated.

Mitochondrial E3 ubiquitin ligase 1 (MUL1) is a mitochondria-located SUMO E3 ligase with an enzymatic activity domain in the C-terminal (RING domain) exposed toward the cytosol 22, 23. Through interacting and transferring mono- or poly-SUMO chains to the SUMO-conjugation motifs of specific substrates, MUL1 plays important roles in cellular mitochondrial homeostasis and inflammasome activation 24-27. Researchers have found that changes in cellular and extracellular conditions affect mitochondrial function through the regulatory activity of MUL1 in mitophagy and mitochondrial dynamics 24, 25. Moreover, MUL1 triggers the innate immune response through SUMOylation of NLRP3 and RIG-1^26^, ^27^ and the pathogenic process in antiviral immunity and inflammatory and neurological diseases 27-29. However, few studies have examined its character in cancer.

In this study, we screened SUMO E3 ligases in published BCa sequencing data (GSE48276) and our own BCa RNA sequencing data (GSE106534) and identified that MUL1 was downregulated in BCa tissues with LN metastasis. Further mechanistic studies revealed that MUL1 SUMOylated HSPA9 and mediated HSPA9 export from mitochondria to the nucleus, resulting in ubiquitination and degradation of SUZ12 and EZH2. In this way, MUL1 inhibited the phosphorylation of STAT3 and prevented BCa cell LN metastasis. Accordingly, our study elucidates the function and underlying mechanism of MUL1 and suggests a novel role of SUMO E3 ligases in LN metastasis of BCa.

## Materials and Methods

### Cell lines and cell culture

The human urinary bladder cancer (BCa) cell lines T24 and UM-UC-3, as well as the human embryonic kidney cell line 293T, were obtained from the American Type Culture Collection (ATCC). The cells were maintained in an incubator with 5% CO_2_ at 37 °C. T24 cells were cultured in RPMI 1640 medium (Gibco), while UM-UC-3 and 293T cells were cultured in DMEM (Gibco). Both media were supplemented with 10% fetal bovine serum (FBS, Sigma-Aldrich) and 1% penicillin-streptomycin (Gibco). Authentication of cell lines was performed, and mycoplasma testing was conducted by IGE Biotechnology (China).

### RNAi and lentivirus transduction

To silence MUL1 in BCa cells, siRNA oligonucleotides targeting MUL1 were designed and synthesized from Suzhou GenePharma Co., Ltd. (China). The sequences of these siRNA oligonucleotides are listed in the Supplemental Table S5. A total of 75 nM siRNA and 5 µl Lipofectamine RNAimax (Life Technologies) were mixed in OptiMEM (GIBCO) to silence MUL1 in BCa cells. Knockdown of EZH2 was performed using the same method as above.

As outlined in our previous publication 30, we followed protocol to establish stable cell lines with knockdown or overexpression of the target gene. 30. The specific siRNA sequences can be found in the Supplemental Table. Briefly, to establish stable MUL1-overexpressing and MUL1-knockdown cells, pCDH-CMV-MCS-EF1-Puro vectors were loaded with the full-length MUL1 sequence. PLKO.1-Puro vectors were loaded with MUL1-siRNA-2 sequences targeting MUL1. Lentivirus production was performed according to our previous study 31. Cell infection with lentivirus was conducted for 48h in the presence of polybrene (IGE BIOTECHNOLOGY LTD, China). Similar methods were performed to establish OE-HSPA9, OE-HSPA9-K612R, OE-HSPA9-K291R and OE-HSPA9-K316R cells. Sequences of wild-type HSPA9 were separately substituted with 'CGA' at 1834-1836 (HSPA9-K612R), 946-948 (HSPA9-K316R) or 871-873 sites (HSPA9-K291R) of coding sequences (CDS) to generate corresponding HSPA9-K612R, HSPA9-K316R or HSPA9-K291R sequences. pCDH-CMV-MCS-EF1-Puro vectors were loaded with wild-type HSPA9, HSPA9-K612R, HSPA9-K291R or HSPA9-K316R sequences.

### *In vitro* migration and invasion assays

Cell migration and invasion abilities were assessed through Transwell assays. Detailed protocols for these assays can be found in our previous publication 6. In the transwell assays, T24 and UM-UC-3 cells were seeded in 8 μm transwell chambers placed in 24-well plates. The lower chambers were supplemented with 700 μl of complete medium. T24 cells were allowed to migrate for 8 hours, while UM-UC-3 cells were incubated for 21 hours. Then, cells in the transwell chambers were fixed with 4% paraformaldehyde (BL539A, Biosharp) for 1 hour and stained with crystal violet (C0121, Beyotime). In the invasion assays, the transwell chambers were preliminarily coated with 25 μg Matrigel (#356234, Corning). T24 cells were incubated for 10 h, and UM-UC-3 cells were incubated for 24 h. Then cells were fixed and stained using the same procedure as in the Transwell assays.

### *In vivo* popliteal lymphatic metastasis model

The mouse popliteal lymphatic metastasis model was established according to our previously described protocols 7, 30, 32. All animal experiments were conducted in compliance with the guidelines and regulations set forth by the Institutional Animal Care and Use Committee of Sun Yat-sen University. Male BALB/c nude mice (3-4 weeks old) were procured from Vital River Laboratory Animal Technology (Beijing, China) and housed under specific pathogen-free (SPF) conditions. Five mice were randomly assigned to each group. Popliteal LNs were dissected after construction of the metastatic model for 4-6 weeks. LN size and volume were measured. Subsequently, the popliteal LNs were embedded in paraffin for subsequent immunohistochemical (IHC) assays and hematoxylin-eosin (HE) staining.

### *In vitro* CCK8 and colony assays

Cell migration and invasion abilities were assessed through CCK8 and colony assays. In the CCK8 assays, a total of 6 × 10^3^ cells were cultured in 96-well plates (#3635, Corning). After addition of 10 μl CCK8 (K1018, APExBIO) for 2.5 h, the OD450 was measured. Colony formation assays were conducted using 1 × 10^3^ T24 or UM-UC-3 cells, which were incubated for a duration of 10 days. Then, the cells were fixed and stained as in the transwell assays.

### Western blotting

Protein lysates were processed using IP lysis buffer (#87788, Thermo Fisher). The resolved proteins were separated by electrophoresis on 10% sodium dodecyl sulfate polyacrylamide gels. Following electrophoresis, the proteins were electrophoretically transferred onto PVDF membranes (IPVH00010, Millipore, USA). The PVDF membranes were blocked with 5% skim milk (P0216, Beyotime) for 1 hour and incubated overnight with the targeted antibodies listed in Supplemental Table S3. After thorough washing with TBST buffer (JXF003, Biosharp), the membranes were incubated with HRP-conjugated anti-rabbit or anti-mouse secondary antibodies (SA00001-1, SA-00001-2). After washing with TBST buffer, bands were immunoblotted using immobilon ECL Ultra Western HRP substrate (WBULS0100, Millipore).

### qRT‒PCR

RNA extraction was performed using an RNA-Quick Purification Kit (RN001, ESscience) following the recommended protocol provided by the manufacturer. The quality and concentration of the isolated RNA samples were assessed through NanoDrop spectrophotometer (Thermo Fisher). Total RNA (0.5 µg) was used to synthesize cDNA with HiScript III RT SuperMix for qPCR (R323-01, Vazyme). qRT‒PCR was then performed to quantify the relative expression levels of specific genes. The primers used for qRT-PCR are provided in the Supplemental Table S4.

### Co-IP and mass spectrometry analysis

For co-IP, IP lysis buffer (#87788, Thermo Fisher) was used to prepare a cellular extract. The cellular extract was incubated with 3.0 µg anti-MUL1, anti-HSPA9, anti-GFP, anti-EZH2 or anti-SUZ12 antibody overnight at 4 °C. Subsquently, the protein complex was linked to protein A/G magnetic beads (HY-K0202, MCE) and incubated for 4 hours. Subsequently, magnetic beads were subjected to five rounds of thorough rinsing with NP40 lysis buffer (P0013F, Beyotime) and resuspended in IP lysis buffer (#87788, Thermo Fisher). Protein interactions with the corresponding primary antibody were detected via immunoblotting.

Mass spectrometry analyses were performed by the Bioinformatics and Omics Center, Sun Yat-Sen Memorial Hospital, Sun Yat-Sen University (Guangzhou, China).

### Cell cycle and apoptosis assays

Cell cycle analysis was conducted following the protocol outlined in a previous study 33. For cell cycle synchronization, 6 × 10^4^ cells were incubated in 6-well culture plates and treated with 4 mM thymidine (T9250, Sigma‒Aldrich) for 16-18 hours. After release with complete medium for 4 hours, a second thymidine treatment was applied for 16-18 hours to induce cell cycle arrest at the G1/S transition. Subsequently, cells were released from the arrest and allowed to progress through the cell cycle for the specified durations (6h for MUL1-silencing cells and 7.5h for MUL1-overexpressing cells). The cells were harvested by centrifugation at 800 rpm for 3 minutes, followed by two washes with PBS. Cold ethanol fixation was then performed at 4 °C for 24 h. Then, the cells were washed twice with PBS (PRS, HyClone) and stained with PI containing RNAase (CCS002, ESscience) for 20 minutes. The distribution of cells across different cell cycle phases were analyzed via flow cytometry (Beckman) and Modfit LT software (Verity Software House). Sync wizard model of Modfit LT software was used to process the results of cell cycle synchronization experiments.

For apoptosis assays, approximately 1 × 10^5^ cells were seeded in 6-well culture plates. The cells were cultured with PBS or 1.5 μM cisplatin (HY-17394, MCE) for 48 hours. After collection and two washes with PBS, the cells were stained with FITC-annexin V and PI (AP001-300, ESscience) for 20 minutes. The percentage of apoptotic cells was analyzed via a flow cytometry (Beckman) and FlowJo VX software (BD Biosciences).

### Immunohistochemistry (IHC)

Tumor tissues embedded in paraffin were carefully sectioned into 5 µm slices. Immunohistochemistry assays were performed following our previous study 6, 34. Tissue sections were stained with anti-MUL1 antibody (16133-1-AP, Proteintech, 1:1000), anti-SUZ12 antibody (20366-1-AP, Proteintech, 1:200), anti-EZH2 antibody (#5246, Cell Signaling Technology, 1:200), anti-p-STAT3 antibody (#9145, Cell Signaling Technology, 1:200), anti-cytokeratin 7 antibody (17513-1-AP, Proteintech, 1:500), anti-CDK1 antibody (19532-1-AP, Proteintech, 1:200) and anti-MMP2 antibody (ab86607, Abcam, 1:200). Representative images were acquired using a Nikon ECLIPSE Ti microscope (Tokyo, Japan). Two experienced pathologists, who were blinded to the experimental conditions, independently assessed the expression levels of MUL1, SUZ12, CK7, EZH2, p-STAT3, CDK1 and MMP2 in specimens. The quantification was performed based on the scoring system previously described in our research 34. Briefly, the staining intensity was categorized into four grades: 0 (negative), 1 (weak), 2 (moderate), and 3 (strong). The H-score, which accounts for both the percentage of positive cells (ranging from 0% to 100%) and the staining intensity, was calculated by multiplying these two factors to term as the staining score.

### Immunofluorescence (IF)

For immunofluorescence assays, cells were incubated in 35 mm confocal dishes (BS-20-GJM, Biosharp). Subsequently, the adherent cells were fixed in 4% paraformaldehyde solution (BL539A, Biosharp) for 15 minutes. To minimize non-specific binding, the cells were then blocked with 5% BSA (ST023, Beyotime) for 1 hour. Following the blocking step, the cells were incubated with the appropriate primary antibody at 4 °C overnight. After washing with PBS (PBS, HyClone), the cells were cultured with a secondary antibody conjugated to fluorescent dye for 1 hour (SA00013-3, SA00013-4, SA00013-2, SA00013-1, Proteintech). DAPI was selectively applied to visualize the cell nuclei. Immunofluorescence images were acquired through a Carl Zeiss microscope (Zeiss) and corresponding Zeiss microscopy software.

### Mitochondrial protein extract

Mitochondrial protein extract assays were performed using a Cell Mitochondria Isolation Kit (C3601, Beyotime). T24 and UM-UC-3 cells were collected up to standard and washed with cold PBS five times. Cells were suspended in 2 ml of cell mitochondria isolation buffer containing 100 mM PMSF (ST506, Beyotime) for 30 minutes. Then, the cell suspension was homogenized with a cell homogenizer (#20060404, YOUYIBO) 30 times on ice. Trypan blue staining (#15250061, GIBCO) was performed to detect the quality of homogenization.

The collected supernatant was subjected to centrifugation at 1000 × g for 10 minutes, followed by an additional centrifugation at 10000 × g for 20 minutes. Then, the precipitate and supernatant fraction were separately collected. The supernatant fraction was centrifuged at 15000 × g for 30 minutes to prepare cytosolic protein lysates with IP lysis buffer (#87788, Thermo Fisher). The precipitation faction was used to prepare mitochondrial protein lysates with mitochondrial protein lysis buffer. For co-IP assays using mitochondrial protein lysates, lysates were prepared with an IP lysis buffer (#87788, Thermo Fisher). A total of 5 × 10^7^ cells were prepared for each assay. For subsequent western blotting assays, TOM70 served as a reference for mitochondrial proteins. For subsequent IF assays, TOM20 served as a reference for mitochondrial location. All centrifugation was performed at 4 °C.

### Nuclear and cytoplasmic protein extracts

We used a nuclear and cytoplasmic protein extraction kit (P0037, Beyotime) to perform this assay. Prior to the extraction of nuclear and cytoplasmic proteins, mitochondrial extraction was performed. The cell suspension was subjected to homogenization using a cell homogenizer, followed by centrifugation at 10000 × g for 20 minutes. The supernatant fraction contained cytoplasmic protein extract buffer A and was incubated for 15 minutes on ice. Then, cytoplasmic protein extract buffer B was added, and the sample was vigorously vortexed for 5 minutes. Subsquently, the lysate was incubated for 1 minute on ice and centrifuged at 13000 × g for 5 minutes. The resulting supernatant fraction, devoid of mitochondria, was carefully collected as the mitochondria-free cytoplasmic protein extract, and the precipitate contained nuclear protein extract buffer. During stationing for 30 minutes, the lysate was vortexed for 2 minutes every 2 minutes. Following centrifugation at 15000 × g for 5 minutes, the supernatant fraction was collected as the nuclear protein lysate. All centrifugation steps were performed at 4 °C.

### GSEA

The Gene Set Enrichment Analysis (GSEA) was conducted using GSEA software in this study. 35, 36. Analysis was based on C6 oncogenic signature gene sets.

### Statistical analyses

Statistical analyses were conducted using SPSS 25.0 software. Statistical analyses were performed according to our previous study 37, 38. Kaplan-Meier survival curves were generated to visualize overall and progression-free survival times, and the log-rank test was utilized to assess statistical significance. Student's t-test was employed to analyze differences between two groups. Pearson correlation analysis was performed to determine the associations between two variables. For multiple variable comparisons, either two-tailed Student's t-test or one-way analysis of variance (ANOVA) was applied. Univariate and multivariate Cox proportional hazards models were employed to estimate hazard ratios (HRs) and 95% confidence intervals (CIs), and to identify independent prognostic factors. All graphs were plotted using GraphPad Prism 9.3.1. Primary data from GEO and TCGA were processed in R studio (R version 4.2.2). The structure model of HSPA9 was predicted and obtained from SWISS-MODEL 39, 40. SUMO-conjugation motifs were predicted using the website tools GPS-SUMO: http://sumosp.biocuckoo.org/online.php; SUMOplot^TM^ Analysis Program: https://www.abcepta.com/sumoplot and JASSA: http://www.jassa.fr/. All significant differences were defined as * *P* < 0.05 or ** *P* < 0.01.

## Results

### MUL1 expression was negatively associated with LN metastasis and poor prognosis in BCa

**Figure 1:**
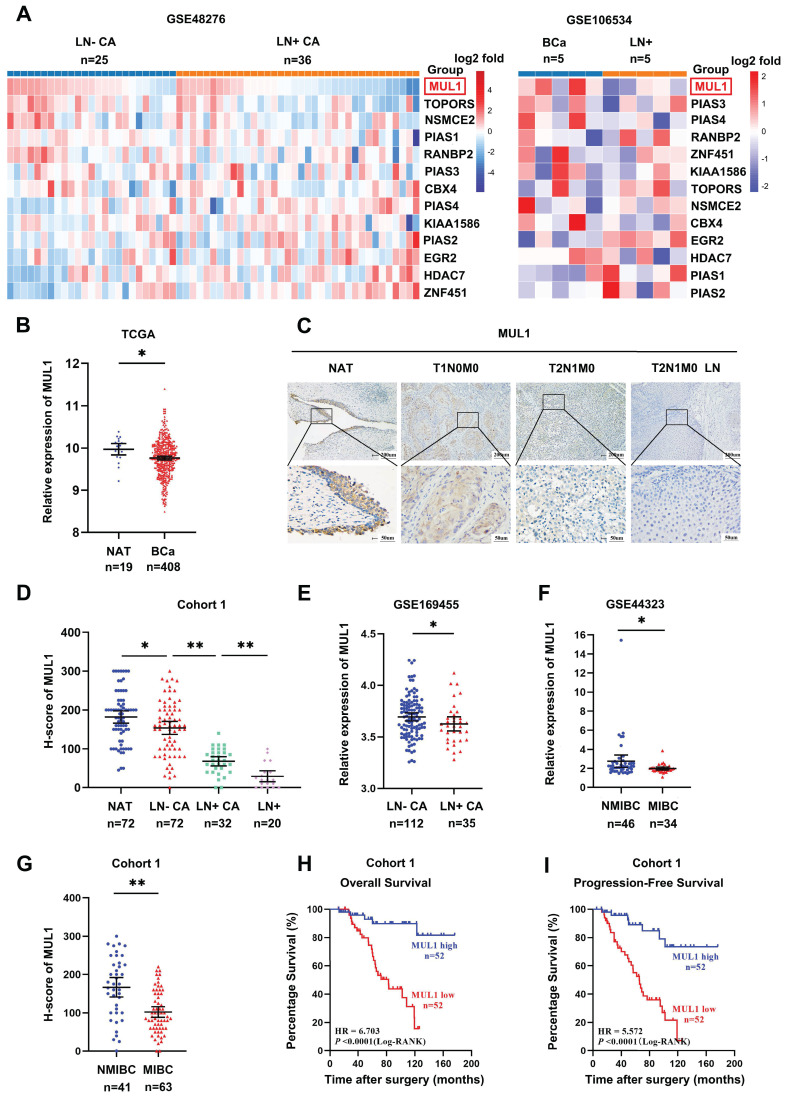
MUL1 expression was negatively associated with LN metastasis and poor prognosis in bladder cancer. (A). Heatmap of the relative expression of SUMO E3 ligases in BCa tissues obtained from GSE48276 and GSE106534. Normalized counts were performed via log2 transformation, and the results are represented by the color intensity scale. (B). Comparison of MUL1 expression in BCa tissues and normal adjacent bladder tissues (NATs) in the TCGA cohort. (C). Protein levels of MUL1 were detected by IHC in NAT, LN- CA, LN+ CA and LN+ in cohort 1. NAT: Normal adjacent tissues; LN+ CA: metastasis-positive BCa tissues; LN-CA: metastasis-negative BCa tissues; LN+: metastatic lymph nodes. (D). The protein level of MUL1 was assessed via IHC in NAT, LN- CA, LN+ CA and LN+ in cohort 1. (E). Comparison of MUL1 expression in LN- CA and LN+ CA tissues in GSE169455. (F-G). Comparison of MUL1 expression in NMIBC and MIBC tissues in GSE44323 (F) and cohort 1 (G). (H-I). Kaplan‒Meier survival analysis of the OS (H) and PFS (I) of BCa patients with high versus low MUL1 levels. One-way analysis of variance (ANOVA) or two-tailed t tests were used to assess statistical significance. *, *P* < 0.05; **, *P* < 0.01.

To identify the SUMO E3 ligases that are most related to LN metastasis in BCa, we first analyzed the expression of 13 SUMO E3 ligases in two BCa datasets. The first dataset included 25 LN metastasis-negative tissues (LN-CA) and 36 LN metastasis-positive tissues (LN+ CA) (GSE48276), while the other dataset included 5 metastasis-positive LN tissues (LN+) paired with the corresponding primary BCa tissues (GSE106534). We observed lower expression of MUL1 in LN+ CA than in LN-CA and a decrease in LN+ compared with paired LN+ CA (Fig. 1A). Meanwhile, we found that MUL1 was downregulated in BCa tissues from The Cancer Genome Atlas (TCGA) BCa cohort (Fig. 1B). Then, we verified the expression of MUL1 via immunohistochemistry (IHC) in 124 BCa tissues (cohort 1) (Fig. 1C). Intriguingly, we found that the expression of MUL1 showed a stepwise decrease from normal adjacent tissues (NATs) to LN- CA, LN+ CA, and LN+ (Fig. 1D). Moreover, we found that MUL1 was downregulated in LN- CA than LN+ CA in GSE169455 (total 149 samples, 2 samples lack of LN metastasis information, thus a total of 147 samples were included in the analysis) (Fig. 1E). Meanwhile, the expression of MUL1 was lower in muscle invasive BCa tissues (MIBC) than in nonmuscle invasive BCa tissues (NMIBC) in three BCa cohorts (GSE44323 and cohort 1) (Fig. 1F-G). Additionally, Kaplan-Meier analysis conducted on cohort 1 demonstrated that patients with low MUL1 expression exhibited a shorter overall survival time (OS) and progression-free survival time (PFS). (Fig. 1H-I). Univariate and multivariate Cox regression analyses showed that high MUL1 expression served as an independent prognostic factor for longer OS and DFS in cohort 1 (Supplemental Table S1-2). Overall, these findings indicate that MUL1 is downregulated in BCa with LN metastasis and may be an underlying prognostic biomarker of BCa.

### MUL1 regulated the translocation of HSPA9 from mitochondria to nucleus

**Figure 2:**
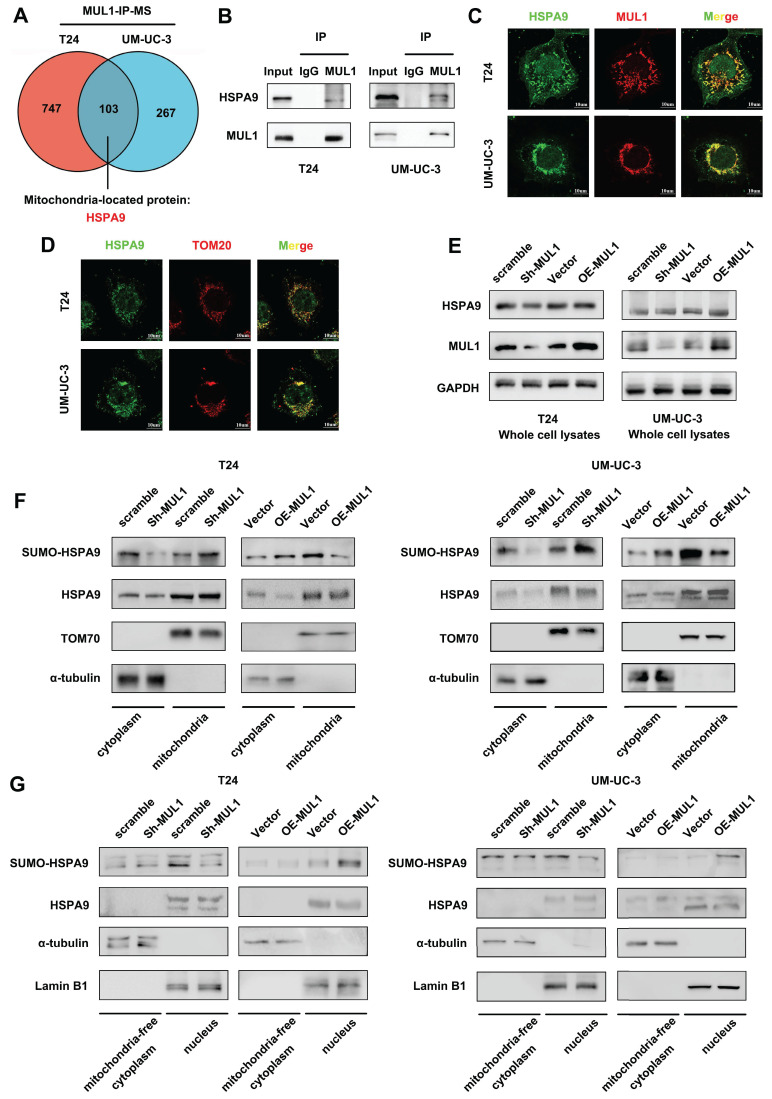
MUL1 regulated the translocation of HSPA9 from mitochondria to nucleus. (A). Identification of MUL1-interacting proteins via MS analysis is shown in Venn diagrams. HSPA9 was one of the 103 overlapping proteins present in both the T24 and the UM-UC-3 MS analysis results. (B). Anti-MUL1 immunoprecipitation (IP) was performed, and HSPA9 was detected via western blotting. Input served as a positive control, and IgG served as a negative control. (C). Representative immunofluorescence (IF) images of MUL1 and HSPA9 colocalization in T24 and UM-UC-3 cells. Green: HSPA9; red: MUL1. Scale bars are shown in the right corner of the images. (D). Representative IF images of TOM20 and HSPA9 colocalization in T24 and UM-UC-3 cells. Green: HSPA9; red: TOM20. Scale bars are shown in the right corner of the images. TOM20 was used as a mitochondrial tracer. (E-F). Mitochondrial, cytosolic and whole-cell lysates of cells with stable MUL1 knockdown or overexpression were prepared. SUMO-HSPA9 and HSPA9 levels were detected via western blotting. TOM70 was used as a reference for mitochondrial proteins, and α-tubulin was used as a reference for cytosolic proteins. (G). Nuclear and mitochondria-free cytoplasmic faction of MUL1-knockdown and -overexpression cells were separated. SUMO-HSPA9 and HSPA9 levels were detected via western blotting. Lamin B1 was used as a reference for nuclear proteins and α-tubulin was used as a reference for mitochondria-free cytoplasmic proteins.

To further investigate the mechanism of MUL1 in LN metastasis, we performed co-immunoprecipitation (co-IP) and mass spectrometry (MS) to identify the primary substrates of MUL1 in T24 and UM-UC-3 cells. Considering that MUL1 was located in the outer membrane of mitochondria, we focused on the mitochondria-located proteins that overlapped in UM-UC-3 and T24 cells according to the MS results (Fig. 2A). We observed that HSPA9 was among the overlapping proteins and was reported to implicated as an important role in oncogenesis 41-43 (Fig. S1A). We further confirmed the interaction of MUL1 and HSPA9 via western blotting and immunofluorescence (IF) assays (Fig. 2B-C). To verify the specificity of MUL1-HSPA9 interaction, we chose another SUMO E3 ligases PIAS1 to detect the potential interaction. As shown in Fig. S1B, we found HSPA9 could not interacted with PIAS1 in BCa cells. Taken together, these results indicate that MUL1 interacted with HSPA9 in BCa cells.

Based on the results above, we firstly analyzed whether MUL1 regulated the expression of HSPA9. As shown in Fig. S1C-D, knockdown or overexpression of MUL1 had no influence on the HSPA9 protein level. Thus, we hypothesized that MUL1 might influence HSPA9 localization. Interestingly, as shown in Fig. 2C-D, HSPA9 could be observed outside mitochondria in BCa cells. We further isolated mitochondrial proteins from T24 and UM-UC-3 cells and found that knockdown of MUL1 decreased HSPA9 level in the mitochondria-free cell fraction (termed cyto) while increasing HSPA9 level in the mitochondrial protein fraction (termed mito). Meanwhile, we observed that SUMOylated HSPA9 level was increased in the mitochondria-free cell fraction and decreased in the mitochondrial protein fraction when MUL1 was overexpressed in T24 and UM-UC-3 cells (Fig. 2E-F). Furthermore, we investigated whether MUL1-mediated HSPA9 translocation mainly located at nucleus. Results showed that knockdown of MUL1 decreased SUMOylated HSPA9 level in nucleus fractions, while overexpression of MUL1 increased SUMOylated HSPA9 level in nucleus fractions. Meanwhile, no significant changes were observed in mitochondria-free cytoplasmic fractions when knockdown or overexpression MUL1 in T24 and UM-UC-3 cells (Fig. 2G). Moreover, we separately performed immunoprecipitation of HSPA9 in mitochondrial and mitochondria-free cell fractions of T24 and UM-UC-3 cells. A higher SUMO2/3 modification level was found in the mitochondria-free cell fraction than in the mitochondrial fraction, which indicated that extramitochondrial HSPA9 had higher SUMOylation level than mitochondrial HSPA9 (Fig. S1E). Collectively, these findings indicate that MUL1 mediated the translocation of HSPA9 from mitochondria to nucleus.

### MUL1 mediated HSPA9 translocation through SUMOylating HSPA9 at the lysine 612 (K612) residue

**Figure 3:**
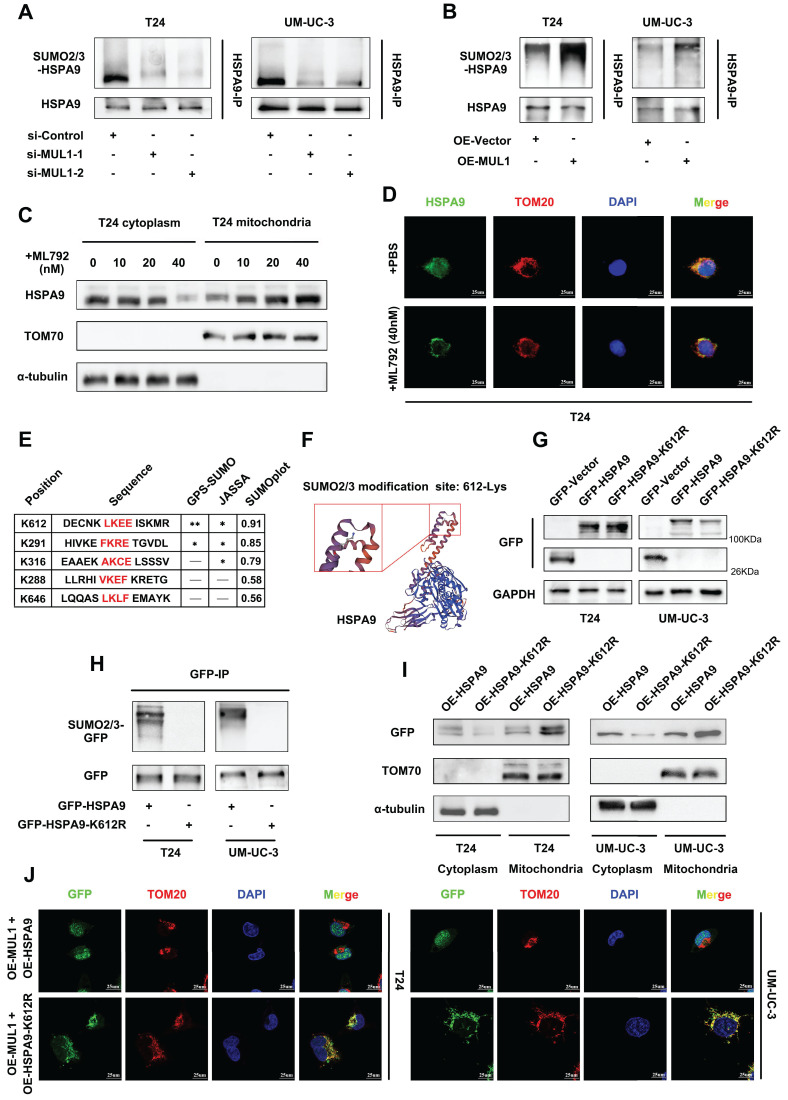
MUL1 SUMOylated HSPA9 at the lysine 612 (K612) residue. (A-B). Anti-HSPA9 IP was performed in MUL1-silencing (E) and MUL1-overexpressing (F) cell lysates. SUMO2/3 and HSPA9 levels were detected via western blotting. (C). T24 cells were treated with ML792 at the concentrations indicated in the images. Mitochondrial and cytosolic cell lysates were separated and prepared. HSPA9 levels in mitochondria and mitochondria-free cell fractions were detected via western blotting. TOM70 was used as a reference for mitochondrial proteins, and α-tubulin was used as a reference for cytosolic proteins. (D). Representative IF images of HSPA9 localization in T24 cells with the treatment of ML792 are shown. Green: HSPA9; red: TOM20; blue: nuclei (DAPI). Scale bars are shown in the right corner of the images. TOM20 was used as a mitochondrial tracer. (E). GPS-SUMO, JASSA and SUMOplot tools were used to predict the HSPA9 SUMOylation site. Sequences around SUMO-conjugation motifs are shown. Red: SUMO-conjugation motifs. (F). Schematic diagram of the predicted HSPA9 K612 site using SWISS-MODEL tools. (G). Wild-type HSPA9 and HSPA9-K612R were overexpressed in T24 and UM-UC-3 cells to generate OE-HSPA9 and OE-HSPA9-K612R cells. Both wild-type HSPA9 and HSPA9-K612R were tagged with GFP. GFP levels were detected via western blotting. (H). Anti-GFP IP was performed in OE-HSPA9 and OE-HSPA9-K612R cell lysates. SUMO2/3 levels were detected via western blotting. (I). Mitochondrial and mitochondria-free cell fractions of OE-HSPA9 and OE-HSPA9-K612R cells were prepared. GFP expression was detected via western blotting. TOM70 was used as a reference for mitochondrial proteins, and α-tubulin was used as a reference for cytosolic proteins. (J). Stable overexpression of exogenous MUL1 was performed in OE-HSPA9 or OE-HSPA9-K612R cells. Both wild-type HSPA9 and HSPA9-K612R were tagged with GFP. Representative IF images of GFP and TOM20 colocalization are shown. Green: GFP; red: TOM20; blue: nuclei (DAPI). Scale bars are shown in the right corner of the images. TOM20 was used as a mitochondrial tracer. -, no statistical significance; *, P < 0.05; **, P < 0.01.

Based on previous studies showing that MUL1 is a SUMO E3 ligase and that SUMOylation is a key factor in inducing protein translocation 20, 21, 23, we hypothesized that MUL1 could mediate the translocation of mitochondrial HSPA9 through SUMOylation. To further confirm our hypothesis, we first investigated whether and how MUL1 SUMOylated HSPA9. As shown in Fig. S2A, we immunoprecipitated HSPA9 from BCa cell extracts and found that HSPA9 was modified by SUMO2/3, but not SUMO1. Additionally, when we used ML792, a specific SUMOylation inhibitor, to treat T24 cells, the SUMOylation level of HSPA9 was decreased according to the concentration of ML792 (Fig. S2B). Furthermore, we detected the SUMOylation of endogenous HSPA9. As shown in Fig. S2C, SUMOylated HSPA9 band was showed at approximate molecular weight of 95KDa (the expected normal size of HSPA9 is 74KDa) and were enhanced after exogenous overexpressing Flag-Ubc9, His-SUMO2 and SUMO3 in 293T cells. Based on these findings, we silenced MUL1 in T24 and UM-UC-3 cells and found a significant decrease in the SUMO2/3 level of HSPA9 (Fig. 3A). Meanwhile, an increase in the HSPA9 SUMO2/3 level was also observed in MUL1-overexpressing BCa cells (Fig. 3B). These results indicate that HSPA9 was SUMOylated by MUL1 in BCa cells in a SUMO2/3-dependent manner.

Then, we used ML792, at different concentrations in T24 cells and found that treatment with higher concentrations of ML792 resulted in higher levels of HSPA9 in mitochondria and lower levels in the mitochondria-free cell fraction (Fig. 3C, S2D). Consistently, we treated T24 and UM-UC-3 cells with ML792 to examine its influence on HSPA9 subcellular location by IF assays. We found that the location of HSPA9 significantly increased in mitochondria, but decreased in nucleus (Fig. 3D, S2E). Treatment of MUL1-overexpressing cells with ML792 markedly increased the HSPA9 level in mitochondria and decreased the HSPA9 level in the mitochondria-free cell fraction (Fig. S2F). These results indicate that MUL1 mediated the translocation of HSPA9 in a SUMOylation-dependent manner.

SUMO-conjugation motifs require the canonical consensus motif ψ-K-X-E (ψ, represents a hydrophobic amino acid, and K is the site of SUMO conjugation) 44. To determine the specific SUMO-conjugation motifs of HSPA9, we performed bioinformatics analysis with the SUMOplot^TM^ Analysis Program, JASSA 45 and GPS-SUMO 46. We identified a SUMOylation site (amino acids 611-614 LKEE) in the HSPA9 amino acid sequence, and K612 lysine residues had the highest score in the analysis (Fig. 3E-F). To examine how HSPA9 was SUMOylated in BCa cells, we separately overexpressed wild-type HSPA9 (termed OE-HSPA9, tagged by GFP) and several HSPA9 site mutants with arginine substitutions (termed OE-HSPA9-K612R, OE-HSPA9-K291R and OE-HSPA9-K316R, all of which were tagged with GFP) in T24 and UM-UC-3 cells (Fig. S3A). We found that only HSPA9-K612R overexpression, neither HSPA9-K291R nor HSPA9-K316R, completely counteracted the SUMOylation of exogenous HSPA9, while MUL1 could also interact with HSPA9-K612R (Fig. 3G-H, Fig. S3B, S3C).

Furthermore, we performed SUMOylation assays to verify the SUMOylation of exogenous overexpressed HSPA9. After overexpressing Flag-Ubc9, His-SUMO2 and SUMO3, GFP-HSPA9 or GFP-HSPA9-K612R in 293T cells, SUMOylated GFP band was showed at approximate molecular weight of 120Kda (the expected normal size of GFP-HSPA9 or GFP-HSPA9-K612R is 95KDa) and were enhanced when we overexpressed GFP-HSPA9 (Fig. S3D). Meanwhile, the SUMOylated GFP band significantly weaken when we overexpressed GFP-HSPA9-K612R (Fig. S3D). These results jointly indicate that HSPA9 is SUMOylated by MUL1 at the K612 site.

Considering that MUL1 mediated the export of HSPA9 from mitochondria by catalyzing the SUMOylation of HSPA9, we investigated whether K612R of HSPA9 could reverse the translocation of HSPA9 induced by MUL1. As shown in Fig. 3I, we found that more exogenous HSPA9 (tagged by GFP) was located in mitochondria in OE-HSPA9-K612R cells than in OE-HSPA9 cells. To further identify whether SUMOylation is essential for the translocation of HSPA9 from mitochondria, we separately overexpressed MUL1 in OE-HSPA9 and OE-HSPA9-K612R cells. We found that a significant portion of GFP was exported from mitochondria when MUL1 was overexpressed in OE-HSPA9 cells, while GFP remained in the mitochondria when HSPA9-K612R was overexpressed (Fig. 3J). We also found that wild-type HSPA9 overexpression led to a higher level of GFP in the nuclear faction rather than the mitochondria-free cytoplasmic faction compared to OE-HSPA9-K612R BCa cells (Fig. S3E). These results indicate that MUL1 regulates the translocation of mitochondrial HSPA9 to the nucleus through HSPA9 SUMOylation at the K612 site.

### SUMOylated HSPA9 catalyzed the degradation of SUZ12 and EZH2

**Figure 4:**
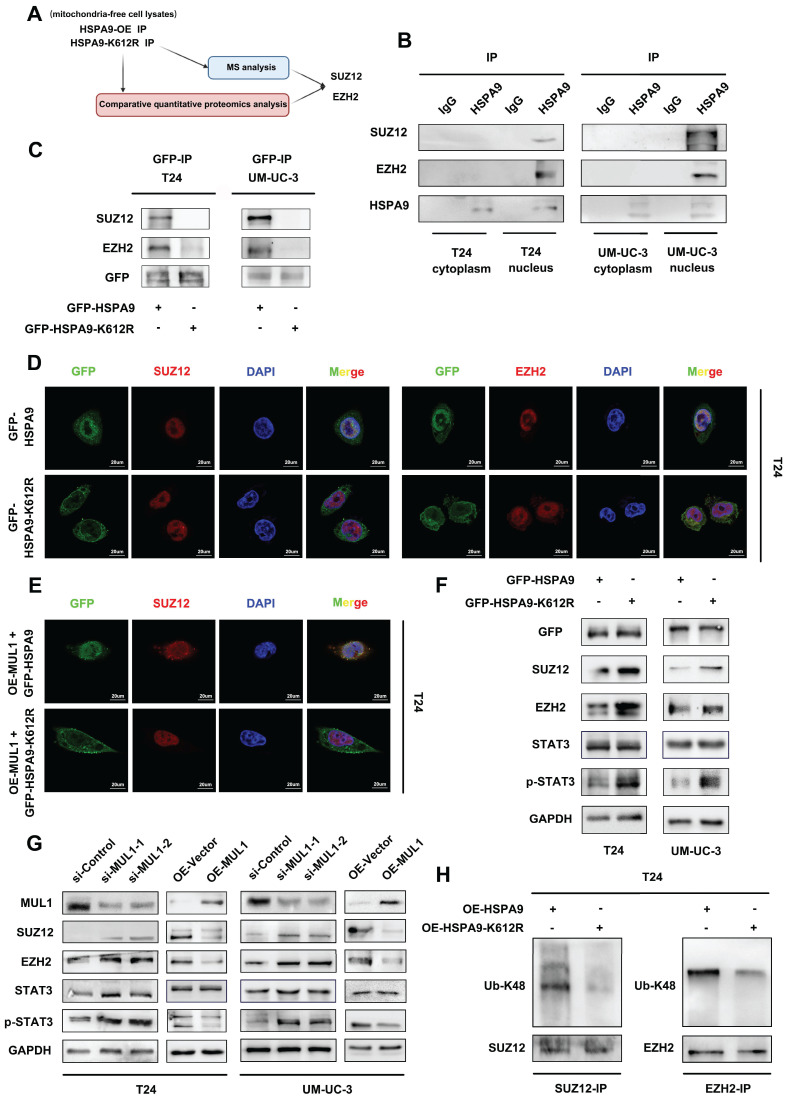
SUMOylated HSPA9 catalyzed the degradation of SUZ12 and EZH2. (A). The identification of HSPA9-interacting proteins is shown in a schematic diagram. By comparing the qualitative MS analysis and relative quantitative proteomics results, SUZ12 and EZH2 were selected for further research. (B). Nuclear and mitochondria-free cytoplasmic faction of T24 and UM-UC-3 cells were separated. Anti-HSPA9 IP was performed in nuclear and mitochondria-free cytoplasmic protein lysates. HSPA9, SUZ12 and EZH2 levels were detected via western blotting. IgG served as a negative control. (C). Anti-GFP IP was performed in OE-HSPA9 and OE-HSPA9-K612R cell lysates. Both wild-type HSPA9 and HSPA9-K612R were tagged with GFP. SUZ12 and EZH2 levels were detected via western blotting. Input served as a positive control, and IgG served as a negative control. (D). Representative IF images of GFP-SUZ12 and GFP-EZH2 colocalization in T24 OE-HSPA9 and OE-HSPA9-K612R cells are shown. Both wild-type HSPA9 and HSPA9-K612R were tagged with GFP. Green: GFP; red: SUZ12/EZH2; blue: nuclei (DAPI). Scale bars are shown in the right corner of the images. (E). Stable overexpression of vector or exogenous MUL1 was performed in T24 OE-HSPA9 and OE-HSPA9-K612R cells. Both wild-type HSPA9 and HSPA9-K612R were tagged with GFP. Representative IF images of GFP and SUZ12 colocalization are shown. Green: GFP; red: SUZ12; blue: nuclei (DAPI). Scale bars are shown in the right corner of the images. (F). SUZ12, EZH2, STAT3 and p-STAT3 levels were detected via western blotting in OE-HSPA9 and OE-HSPA9-K612R cells. Both wild-type HSPA9 and HSPA9-K612R were tagged with GFP. (G). SUZ12, EZH2, STAT3 and p-STAT3 levels were detected via western blotting in MUL1-silencing or MUL1-overexpressing cells. (H). Anti-EZH2 and anti-SUZ12 IP were performed in T24 OE-HSPA9 and OE-HSPA9-K612R cell lysates. Ubiquitin-K48 levels were detected via western blotting.

To further elucidate the role of translocated HSPA9 in BCa cells, co-IP and MS assays (qualitative and comparative quantitative proteomics analysis) using mitochondria-free cell lysates were conducted to compare OE-HSPA9 and OE-HSPA9-K612R and to explore the binding proteins of HSPA9. (Fig. 4A, S4A-B). Through gene set enrichment analysis (GSEA), we observed that signaling pathways related to polycomb repressive complex 1 (PRC1) and polycomb repressive complex 2 (PRC2) were among the most significantly enriched pathways (Fig. S4C-D). Considering that many studies have reported that PRC2 promotes LN metastasis 47 and that an essential subunit of PRC2, SUZ12, was found in our MS analysis results, we selected SUZ12 and another core component of PRC2, EZH2, for further investigation. As shown in Fig. S5A and B, HSPA9 interacted with SUZ12 and EZH2 in BCa cells. However, we found that neither SUZ12 nor EZH2 bound to MUL1 (Fig. S5C-D). Subsequently, considering that the K612 site was found to be essential for HSPA9 localization, we examined the subcellular location of HSPA9-SUZ12 and HSPA9-EZH2 interactions. We separately performed immunoprecipitation of HSPA9 in mitochondria-free cytoplasmic and nuclear fractions of T24 cells. We found that HSPA9-SUZ12 and HSPA9-EZH2 interaction occurred in nuclear fractions (Fig. 4B). Additionally, we found that exogenous HSPA9 interacted with SUZ12 and EZH2 in the nucleus of OE-HSPA9 BCa cells but disappeared in OE-HSPA9-K612R cells (Fig. 4C-D, Fig. S5E). Meanwhile, we immunoprecipitated HSPA9 in MUL1-silencing cells and found that knockdown of MUL1 largely weakened both the GFP-SUZ12 and GFP-EZH2 interactions in BCa cells (Fig. S5F). Moreover, as shown in Fig. 4E and S5G, MUL1 overexpression markedly increased GFP-SUZ12 colocalization in the nucleus of OE-HSPA9 BCa cells, which was not observed in OE-HSPA9-K612R BCa cells. Taken together, these results indicate that SUMOylated HSPA9 interacts with SUZ12 and EZH2 in the nucleus, while mutation of the K612 site prevents the interactions by inhibiting HSPA9 mitochondrial translocation.

Previous studies have elucidated that the STAT3 signaling pathway, which is a key epigenetic factor in LN metastasis and BCa progression, can be activated by EZH2 48, 49. Based on these findings, we hypothesized that the STAT3 signaling pathway might be downstream of SUMOylated HSPA9 in BCa cells. Firstly, we demonstrated that knowndown of EZH2 increased the level of p-STAT3, while had no effect on the level of STAT3 in BCa cells (Fig. S5I). Then we investigated whether SUMOylated HSPA9 could affect the stability of SUZ12 or EZH2 and the activation of STAT3. As shown in Fig. 4F, significantly decreased levels of SUZ12, EZH2, and p-STAT3 were observed in OE-HSPA9 cells compared to OE-HSPA9-K612R cells. Moreover, we found that knockdown of MUL1 increased the level of SUZ12, EZH2 and p-STAT3, while decreased levels of SUZ12, EZH2 and p-STAT3 were observed in MUL1-overexpressing BCa cells (Fig. 4G). We further explored the mechanism by which HSPA9 induced SUZ12 and EZH2 degradation. Previous studies have demonstrated that the degradation of PRC2 occurs in a ubiquitin-K48-dependent manner 50. Therefore, we detected the Ub-K48 levels of SUZ12 and EZH2 using immunoprecipitation in T24 and UM-UC-3 cells. Compared to OE-HSPA9 cells, the Ub-K48 modification of SUZ12 and EZH2 was significantly decreased in OE-HSPA9-K612R cells. (Fig. 4H, S5H). These results reveal that SUMOylated HSPA9 induces the degradation of SUZ12 and EZH2 in a ubiquitin-K48-dependent manner.

As demonstrated in previous studies, activated STAT3 promotes cancer cell metastasis through transcriptional activation of multiple mediators, including MMP2, MMP9 and EMT-related genes 51-53. Thus, we investigated the expression level of STAT3 downstream metastasis-related genes. We found that knockdown of MUL1 increased the transcriptional level of MMP2, MMP9, TWIST and N-cadherin, while decreased the level of E-cadherin (Fig. S6A). Consistently, overexpression of MUL1 decreasing the level of MMP2, MMP9, TWIST and N-cadherin, while increased the level of E-cadherin (Fig. S6B). Then we investigated the influence of K612R on these STAT3 downstream genes. Compared to OE-HSPA9 cells, HSPA9-K612R cells had higher level of MMP2, MMP9, TWIST and N-cadherin, while a lower level of E-cadherin (Fig. S6C). Meanwhile, we detected the expression of STAT3 downstream proliferation-related genes, including cyclin B1, CDK1, c-myc and P21, as have been demonstrated as regulator of G2/M cell cycle arrest in previous study 54-57. Increased level of cyclin B1, CDK1 and c-myc was observed in MUL1-silencing cells, while decreased level of P21 was observed in MUL1-silencing cells (Fig. S7A). We also found decreased level of cyclin B1, CDK1 and c-myc in MUL1-overexpressed cells, while increased level of P21 in MUL1-overexpressed cells (Fig. S7B). Similarly, we found increased level of cyclin B1, CDK1 and c-myc and decreased level of P21 in OE-HSPA9-K612R cells compared to OE-HSPA9 cells (Fig. S7C). Overall, these results showed that MUL1 downregulated the transcriptional expression of STAT3 downstream metastasis-related genes and proliferation-related genes.

### MUL1 suppressed BCa cell migration and invasion *in vitro* and LN metastasis *in vivo*

**Figure 5:**
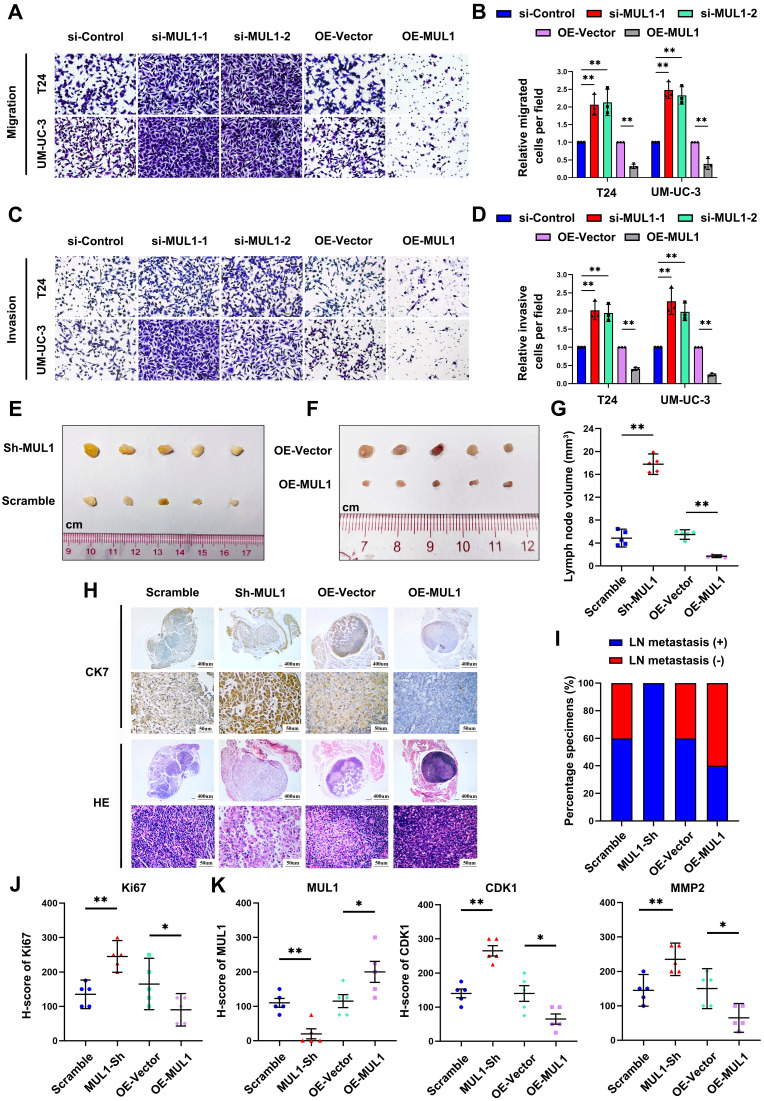
MUL1 suppressed BCa cell migration and invasion *in vitro* and LN metastasis *in vivo*. (A-D). Representative images of migration (A) and invasion (C) assays of MUL1-silencing or MUL1-overexpressing cells. The number of migratory (B) and invasive (D) cells in migration assays was measured and is shown in the histogram. (E-F). Representative images of metastatic LNs in each group (n=5 per group). (G). Statistical analysis of LN volumes in each group (n=5 per group) shown in a histogram. (H-I). LN status percentages were confirmed and measured by H.E. staining and anti-CK7 IHC (H). A histogram was constructed to show LN status percentages among groups (I). (J). Statistical analysis of Ki67 level among groups. Expression level of Ki67 were measured by IHC. (K). Statistical analysis of MUL1, CDK1 and MMP2 expression among groups. Expression level of MUL1, CDK1 and MMP2 were measured by IHC. One-way analysis of variance (ANOVA) or two-tailed t tests were used to assess statistical significance. The standard deviations of three independent experiments are represented by error bars. *, *P* < 0.05; **, *P* < 0.01.

Considering the regulatory role of MUL1 in the HSPA9-PRC2-STAT3 axis, we next examined the characteristics of MUL1 in BCa cells *in vitro.* We used two independent small interfering RNAs (siRNAs) (termed si-MUL1-1 and si-MUL1-2) to knockdown MUL1 in BCa cells (Fig. S1C-D). As shown in Fig. 5A-D, silencing MUL1 markedly promoted the migration and invasion of BCa cells (Fig. 5A-D). Then, we stably silenced MUL1 in BCa cells with short hairpin RNA (shRNA) (Fig. S8A-B). Consistently, we found that stable inhibition of MUL1 promoted the migration and invasion of BCa cells (Fig. S8C-E). Subsequently, we stably overexpressed MUL1 in T24 and UM-UC-3 cells (Fig. S1C-D) and found that MUL1 overexpression remarkably suppressed the migration and invasion of BCa cells (Fig. 5A-D). These findings confirm that MUL1 can inhibit the migration and invasion of BCa cells *in vitro*.

To evaluate whether MUL1 is involved in LN metastasis of BCa cells *in vivo*, we generated a popliteal LN metastasis model by injecting MUL1-silencing or MUL1-overexpressing UM-UC-3 cells into the footpads of BALB/c mice. We found that the volumes of popliteal LNs in the MUL1-knockdown group were larger than the control group, while the volumes of popliteal LNs in the MUL1-overexpressing group were much smaller (Fig. 5E-G). IHC staining analysis of the epithelial cell marker cytoskeletal 7 (CK7) was performed to confirm LN metastasis (Fig. 5H). In the MUL1-knockdown group, the rate of LN metastasis significantly increased from 60% in the control group to 100%, whereas in the MUL1-overexpressing group, the rate decreased from 60% in the vector group to 40% (Fig. 5I). In order to detect the proliferative capacity of the tumors among different groups, we examined the Ki67 staining of the footpad tumors by IHC assays. In the MUL1-knockdown group, the H-score of Ki67 increased compared to the scramble group, while in the MUL1-overexpressing group, the the H-score of Ki67 decreased compared to the vector group (Fig. 5J, S9A). Furthermore, we detected the expression of CDK1 and MMP2 among different groups in tumors of the footpad by IHC staining analysis. Compared to the corresponding control group, we found that the expression of CDK1 and MMP2 decreased in MUL1-overexpression group, while upregulated in MUL1-knowdown group (Fig. 5K, S9B). Collectively, these findings demonstrate that MUL1 can inhibit BCa cell LN metastasis *in vivo*.

### MUL1 inhibited BCa cell proliferation by arresting cell cycle progression in the G2/M phase

**Figure 6:**
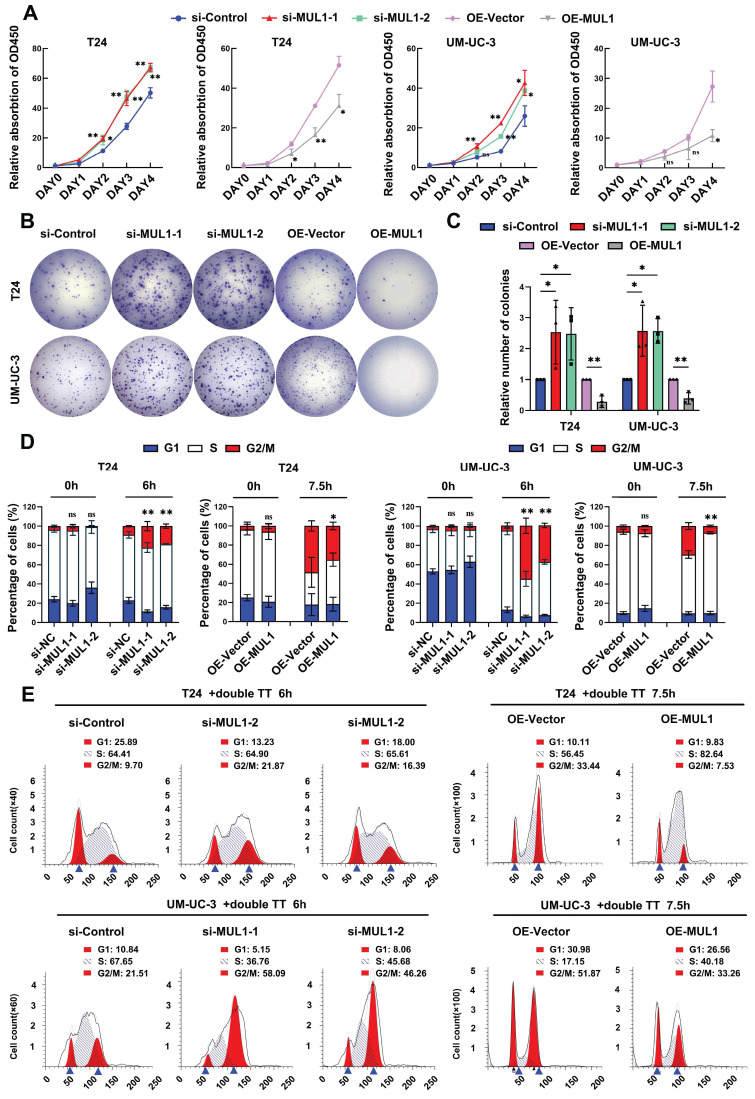
MUL1 inhibited BCa cell proliferation by arresting cell cycle progression in the G2/M phase. (A). Viability curves of MUL1-silencing or MUL1-overexpressing cells were constructed according to CCK8 assay results. (B-C). Colony formation assays were performed with MUL1-silencing or MUL1-overexpressing T24 and UM-UC-3 cells (B). Statistical analyses of colony numbers in colony formation assays are shown in the histogram (C). (D). Cell cycle synchronization assays were performed with MUL1-silencing or MUL1-overexpressing T24 and UM-UC-3 cells. Histograms were plotted to show the percentages of the cell populations at different cell cycle stages. (E). Representative images of T24 cell cycle synchronization assays. T24 MUL1-silencing or MUL1-overexpressing cells were blocked with double thymidine. Cells were released for 6 or 7.5 hours after double thymidine blocks. Then, cells before and after release were collected to perform cell cycle assays. The X axis represents PE-A channels, and the Y axis represents cell counts. One-way analysis of variance (ANOVA) or two-tailed t tests were used to assess statistical significance. The standard deviations of three independent experiments are represented by error bars. *, *P* < 0.05; **, *P* < 0.01.

Next, by performing CCK8 and colony formation assays, we investigated whether MUL1 could suppress the proliferation of BCa cells. We observed that MUL1 silencing markedly promoted the viability and colony formation capacity of BCa cells (Fig. 6A-C), while overexpressing MUL1 dramatically inhibited BCa cell proliferation (Fig. 6A-C). We also found that stable inhibition of MUL1 accelerated BCa cell proliferation (Fig. S10A-C). We further investigated whether MUL1 is involved in the regulation of BCa cell proliferation by halting the cell cycle progression or triggering cell apoptosis. As shown in Fig. S8A-C, no difference in the proportion of apoptotic cells was observed in BCa cells after MUL1 silencing or overexpression (Fig. S11A-C). These results led us to further investigate whether MUL1 participates in cell cycle arrest.

Therefore, we performed cell cycle synchronization assays to explore the character of MUL1 in regulating cell cycle progression in BCa cells. We blocked cell cycle progression in T24 and UM-UC-3 cells at the G1/S phase via the dithymidine block method. After treatment with double thymidine, BCa cells were released for 6 h (MUL1-silencing cells) or 7.5 h (MUL1-overexpressing cells) in naïve cultures. We observed an increase in the proportion of cells in the G2/M phase in MUL1-silenced cells compared to the control cells. Meanwhile, the percentage of MUL1-overexpressing cells in the G2/M phase was significantly lower. (Fig. 6D-E, S11D). In conclusion, MUL1 inhibits the proliferation of BCa cells through inducing G2/M-phase cell cycle arrest.

### MUL1-mediated inhibition of BCa metastasis and proliferation was dependent on the SUMOylation of HSPA9

**Figure 7:**
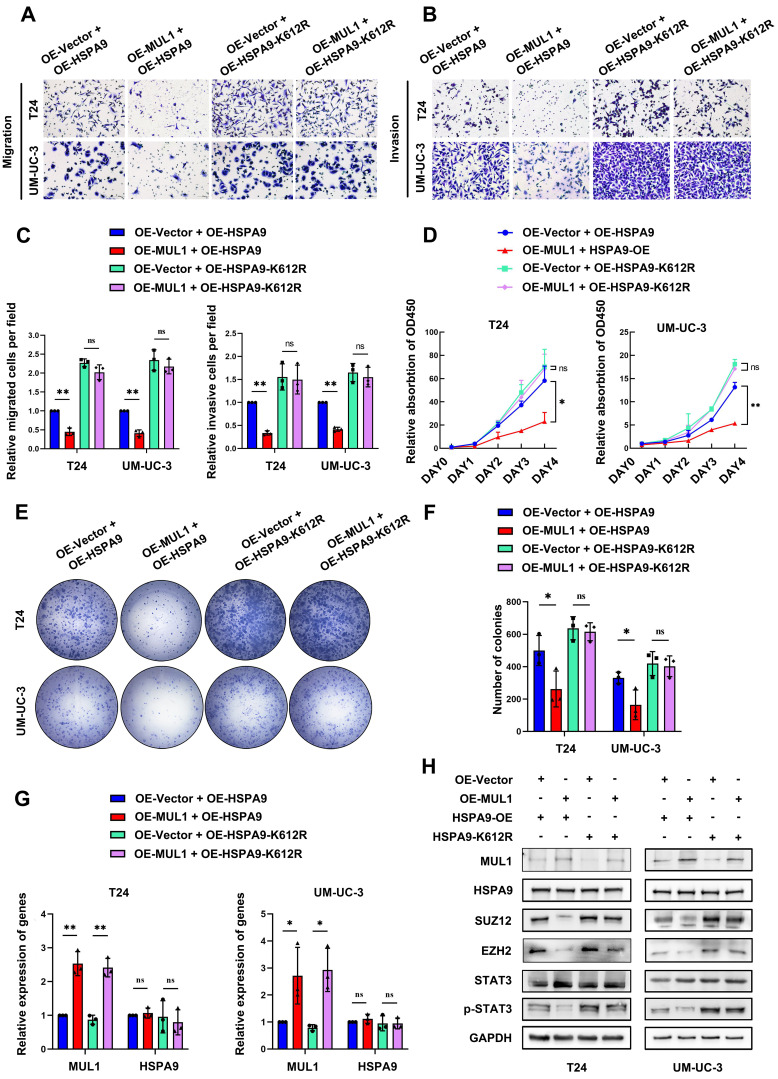
MUL1 inhibited BCa progression, and this activity was dependent on the SUMOylation of HSPA9. (A-C). Representative images of migration (A) and invasion (B) assays that explored MUL1 function in OE-HSPA9 and OE-HSPA9-K612R cells. MUL1 or vector was stably overexpressed in OE-HSPA9 or OE-HSPA9-K612R cells. The numbers of migratory and invasive cells are shown in the histogram (C). (D). CCK8 assays were performed in OE-HSPA9 and OE-HSPA9-K612R cells with MUL1 or vector overexpression. Viability curves were constructed using relative multiples of the relative absorption at 450 nm. (E-F) Representative images of colony formation assays that explored MUL1 function in OE-HSPA9 and OE-HSPA9-K612R cells (E). The number of colonies was measured and is shown in the histogram (F). (G). The overexpression efficiency of MUL1 and HSPA9 was detected by qPCR in OE-HSPA9 and OE-HSPA9-K612R cells with MUL1 or vector overexpression. (H). MUL1, HSPA9, SUZ12, EZH2, STAT3 and p-STAT3 levels in OE-HSPA9 and OE-HSPA9-K612R cells overexpressing MUL1 or vector were detected via western blotting. One-way analysis of variance (ANOVA) or two-tailed t tests were used to assess statistical significance. The standard deviations of three independent experiments are represented by error bars. *, *P* < 0.05; **, *P* < 0.01.

We further explored whether SUMOylated HSPA9 was essential for the suppressive effects of MUL1. We separately overexpressed HSPA9 and HSPA9-K612R in MUL1-overexpressing BCa cells. As shown in Fig. 7A-G, HSPA9 overexpression remarkably suppressed the migration, invasion and proliferation abilities of OE-MUL1 cells. However, the suppressive effect of MUL1 overexpression was significantly diminished in OE-MUL1 cells overexpressing HSPA9-K612R. Consistent with the above results, decreased levels of SUZ12, EZH2 and p-STAT3 were observed when HSPA9 was overexpressed in OE-MUL1 cells, while no significant changes were observed when HSPA9-K612R was overexpressed (Fig. 7H). Then we investigated the transcriptional level of STAT3 downstream metastasis-related genes among these groups. Results revealed that decreased levels of MMP2, MMP9, TWIST and N-cad and increased level of E-cad were observed when overexpressed MUL1 in OE-HSPA9 cells, while no significance was observed in OE-HSPA9-K612R BCa cells (Fig. S12A). Consistently, decreased level of cyclin B1, CDK1, c-myc and increased level of P21 was observed when overexpressed MUL1 in OE-HSPA9 cells, while no significance was observed when overexpressed MUL1 in OE-HSPA9-K612R cells (Fig. S12B). These results demonstrate that MUL1 exerts tumor-inhibitory effect in BCa cells and that this activity is dependent on the SUMOylation of HSPA9.

In view of our findings indicating the activation of STAT3 signaling pathway through the degradation of SUZ12 and EZH2 mediated by SUMOylated HSPA9, we investigated the role of STAT3 in MUL1-induced tumor inhibition effects. We silenced STAT3 in MUL1-silencing BCa cells (Fig. S13A). Through transwell assays, we found that STAT3 knockdown counteracted the promotion of migration and invasion induced by MUL1 knockdown (Fig. S13B-C). Similarly, we observed that STAT3 knockdown abrogated the proliferation induced by MUL1 knockdown (Fig. S13D-F). Then we examined the transcription level of STAT3 downstream metastasis-related genes among groups. We found that STAT3 knockdown significantly counteracted the change of transcriptional level of MMP2, MMP9, TWIST, N-cad, E-cad, cyclin B1, CDK1, c-myc and P21 induced by MUL1 knockdown (Fig. S14A-B). Taken together, these data indicate that MUL1 SUMOylated HSPA9 and induced PRC2 degradation, thereby impeding the STAT3 signaling pathway to inhibit BCa progression.

### Clinical relevance of the MUL1-PRC2-STAT3 axis in BCa tissues

**Figure 8:**
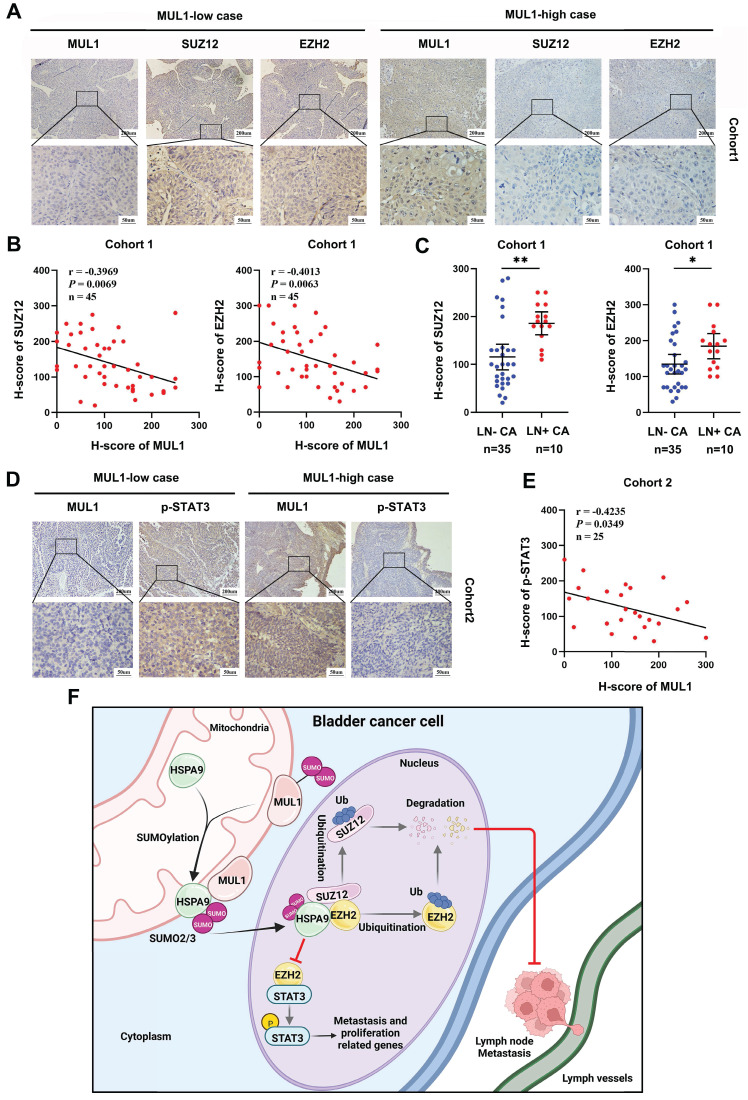
Clinical relevance of the MUL1-PRC2-STAT3 axis in BCa tissues. (A). The EZH2 and SUZ12 protein levels were detected by IHC in cohort 1. (B). Scatter diagrams were plotted to show Pearson correlations between the protein levels of MUL1 and EZH2 or SUZ12 in cohort 1. (C). Comparisons of SUZ12 and EZH2 levels in LN- CA and LN+ CA in cohort 1. (D). The protein level of p-STAT3 was detected via IHC in cohort 2. (E). A scatter diagram was plotted to show Pearson correlations between the protein levels of MUL1 and p-STAT3 in cohort 2. (F). A schematic diagram showing the molecular mechanism of MUL1 in this study. LN+ CA: metastasis-positive BCa tissues; LN-CA: metastasis-negative BCa tissues. One-way analysis of variance (ANOVA) or two-tailed t tests were used to assess statistical significance. *, *P* < 0.05; **, *P* < 0.01.

To further validate the role of the MUL1-HSPA9-PRC2 axis in BCa progression, we performed IHC to evaluate the protein expression relationship between MUL1 and SUZ12 and EZH2 in cohort 1. We observed a significant negative correlation between the expression of MUL1 and EZH2 or SUZ12 (Fig. 8A-B). Meanwhile, increased levels of SUZ12 and EZH2 were observed in LN+ CA compared with LN- CA (Fig. 8C).

We next explored the connection between MUL1 and p-STAT3 in another clinical cohort (termed cohort 2) consisting of 25 BCa tissues obtained from Sun Yat-Sen Memory Hospital. We found that the protein level of p-STAT3 was negatively correlated with that of MUL1 (Fig. 8D-E). Overall, these findings elucidate that MUL1 is negatively correlated with PRC2 and p-STAT3 in clinical BCa cases.

## Discussion

Mitochondria are the main sites that generate biological energy during cancer metastasis. In recent years, accumulating studies have demonstrated that the crosstalk between mitochondria and other subcellular compartments has a critical impact on mitochondrial homeostasis and the intracellular epigenetic state and that the translocation of key molecules is an essential step in this process 58-60. However, whether and how mitochondrial proteins are exported into the cytoplasm and nucleus remain largely unknown. In our study, we found that MUL1 mediates the export of mitochondrial HSPA9 through SUMOylation of HSPA9 at the K612 site. The translocation of SUMOylated HSPA9 from mitochondria to the nucleus induced the degradation of SUZ12 and EZH2, which suppressed the activation of STAT3 signaling and ultimately prevented the LN metastasis of BCa cells (Fig. 8F). Taken together, these findings contribute to a more comprehensive understanding of the mitochondrial protein translocation process and highlight the importance of mitochondrial proteins in LN metastasis.

In contrast to other SUMO E3 ligases, MUL1 is located at the mitochondrial outer membrane and has both SUMO E3 ligase and ubiquitin E3 ligase enzymatic activity 22, 23. As a SUMO E3 ligase, MUL1 catalyzes SUMO1 modification of Drp1 and activates BAX to stabilize the ER/mitochondria platform to facilitate cell death 24. Another study revealed that MUL1 suppresses the activation of NLRP3 by mediating SUMO2/3 modification 26. MUL1 also regulates the inflammatory response to dsDNA through SUMOylation of RIG-1^27^. However, no evidence indicating that MUL1 is involved in LN metastasis has been reported. In our study, we demonstrated the suppressive role of MUL1 in LN metastasis of BCa. In addition, we observed downregulation of MUL1 in BCa cases with LN metastasis. Low MUL1 expression emerged as an independent factor of poor prognosis in BCa patients.

SUMOylation has been reported to participate in the mitochondrial import process and protein quality control 61, 62. For instance, SUMOylation of the fusion protein SYNJ2BP-COX16 induces its translocation to mitochondria to enhance mitochondrial fission in breast cancer 21. Increased SUMOylation levels of mitochondrial proteins were also observed in cells with impaired proteostasis or failed mitochondrial import 62. In this study, we found that MUL1 catalyzes SUMO2/3 modification of HSPA9 at the K612 site but not in a ubiquitin- or SUMO1-dependent manner. Meanwhile, SUMO2/3 modification induced the export of mitochondrial HSPA9 rather than affecting its expression. Similar to our results, a previous study revealed that Cargo-selected transport from mitochondria to peroxisomes was facilitated by the MUL1-mediated formation of mitochondria-derived vesicles (MDVs) 63. Therefore, we hypothesized that SUMOylated HSPA9 forms MDVs during translocation to the nucleus, but further electron microscopy experiments are needed to validate this hypothesis.

HSPA9 is a member of the HSP70 subfamily, which occupies an essential position as a molecular chaperone in multiple cellular physiological activities 64. Human mitochondrial HSPA9 was demonstrated to play a vital role in mitochondrial homeostasis and was found to be correlated with several mitochondria-related diseases 64, 65. For instance, the IP3R1-HSPA9-VDAC1 axis participates in mitochondrial oxidative stress in diabetic atrial remodeling 66. HSPA9 was reported to maintain MEK-ERK-driven tumor cell survival through inhibiting ANT3-mediated mitochondrial membrane permeability 67. Interestingly, HSPA9 is regarded as a proto-oncogene and is aberrantly overexpressed in several cancer types. In prostate cancer, HSPA9 transfers NKX3.1 to mitochondria and confers protection against oxidative stress 43. In breast cancer, HSPA9 contributes to cancer metastasis by mediating epithelial-mesenchymal transition (EMT) 68. Intriguingly, as shown in our results, HSPA9-K612R counteracted the suppressive effect of MUL1 in BCa cells, while no influence on the suppressive effect of MUL1 was observed after HSPA9 overexpression. These results revealed that mitochondrial HSPA9 might promote cancer progression. However, by inducing the translocation of mitochondrial HSPA9 to the nucleus, MUL1-induced SUMOylation of HSPA9 played a tumor suppressive role in BCa cells. Our findings illustrate that HSPA9 is a double-edged sword in LN metastasis and that the translocation of mitochondrial molecules might reverse its original function.

As a notable epigenetic driver, EZH2 is responsible for trimethylation of histone H3 at lysine 27 (H3K27me3) and mono-ubiquitinylates histone H2A at lysine 119 (H2AK119Ub1) 69. SUZ12, EZH2 and other components, such as EED, form PRC2 to mediate broad H3K27me3 and H2AK119Ub1^69^. In addition to the classic function of EZH2 as a histone methyltransferase, EZH2 also regulates the activation of target proteins, such as STAT3, through PRC2-independent nonhistone methylation 70, 71. Meanwhile, the recruitment of active STAT3 to the promoter of PRC2 subunits preserves the expression of SUZ12, EZH2 and other PRC2 subunits 72, 73, while the degradation of SUZ12 and EZH2 is considered to be catalyzed in a ubiquitination-proteasome-dependent manner 74. In various tumors, including bladder cancer, PRC2 has been identified as a key factor promoting tumor progression and LN metastasis. Detecting urinary tumor-secreted cf-EZH2 serves as a diagnostic marker for predicting BCa patients' prognosis and distinguishes MIBC from NMIBC 75-78. Moreover, SUMO E3 ligase CBX4 and PIASXß have been reported to directly SUMOylate EZH2 and enhance its H3K27 methyltransferase activity 79, 80. Herein, we demonstrated that HSPA9 regulates the stability of SUZ12 and EZH2 in a SUMO2/3-dependent manner. The HSPA9-SUZ12 and HSPA9-EZH2 interactions induced ubiquitination-mediated proteasomal degradation of SUZ12 and EZH2, while mutation of the HSPA9 K612 site blocked this process. Overall, our study demonstrated a novel character of SUMOylation in the regulation of PRC2, suggesting that targeting the balance between SUMOylation and deSUMOylation of critical molecules might be a new strategy for treating LN-metastatic BCa.

In conclusion, we demonstrated that the SUMO E3 ligase MUL1 suppresses LN metastasis and proliferation in BCa by mediating SUMOylation of HSPA9. Elucidating the precise function of the SUMO E3 ligase MUL1 in BCa would enable the development of an innovative therapeutic approach for BCa patients with LN metastasis.

## Supplementary Material

Supplementary figures and tables.
