# Supplementary material for: SUMO E3 ligase MUL1 inhibits lymph node metastasis of bladder cancer by mediating mitochondrial HSPA9 translocation
Source: Int J Biol Sci. 2024 Jul 15;20(10):3986–4006. doi: 10.7150/ijbs.98772 (PMC11302872; doi:10.7150/ijbs.98772)
Supplement: Supplementary file 1 — Supplementary figures and tables. [file ijbsv20p3986s1.pdf]

Supplemental Materials

Table of Contents

Supplemental Figure S1: MUL1 interacted with HSPA9.. 3

Supplemental Figure S2: MUL1 mediated the translocation of HSPA9 through SUMOylation.. 5

Supplemental Figure S3: MUL1 catalyzed SUMOylation of HSPA9 at K612 site. 7

Supplemental Figure S4: Mutation of HSPA9 K612 site was relevant to PRC2 signaling pathway. 9

Supplemental Figure S5: SUMOylated HSPA9 interacted and induced the degradation of SUZ12 and EZH2. 10

Supplemental Figure S6: Stable knockdown of MUL1 promoted metastasis-related genes. 12

Supplemental Figure S7: Stable knockdown of MUL1 promoted proliferation-related genes 13

Supplemental Figure S8: Stable knockdown of MUL1 promoted migration and invasion of BCa cells. 14

Supplemental Figure S9: MUL1 inhibited bladder cancer proliferation and LN metastasis *in vivo*. 15

Supplemental Figure S10: Stable knockdwn of MUL1 promoted proliferation of BCa cells. 16

Supplemental Figure S11: MUL1 mediated G2/M phase arrest in BCa.. 17

Supplemental Figure S12: Mutation of HSPA9 K612 site reversed MUL1-induced inhibition of STAT3 downstream metastasis-related genes and proliferation-related genes. 19

Supplemental Figure S13: Inhibition of STAT3 signaling reversed MUL1-induced tumor suppressive effects in BCa. 20

Supplemental Figure S14: Inhibition of STAT3 reversed MUL1-induced inhibition of STAT3 downstream metastasis-related genes and proliferation-related genes. 22

Supplemental Table S1: Univariate and multivariate analysis of factors associated with overall survival in cohort1. 23
Supplemental Table S2: Univariate and multivariate analysis of factors associated with progression free survival in cohort1. 23
Supplemental Table S3: Antibody used in this study. 23
Supplemental Table S4: Primers used in this study. 25
Supplemental Table S5: Sequences of siRNAs and shRNAs in this study. 25

Supplemental Figure Legend

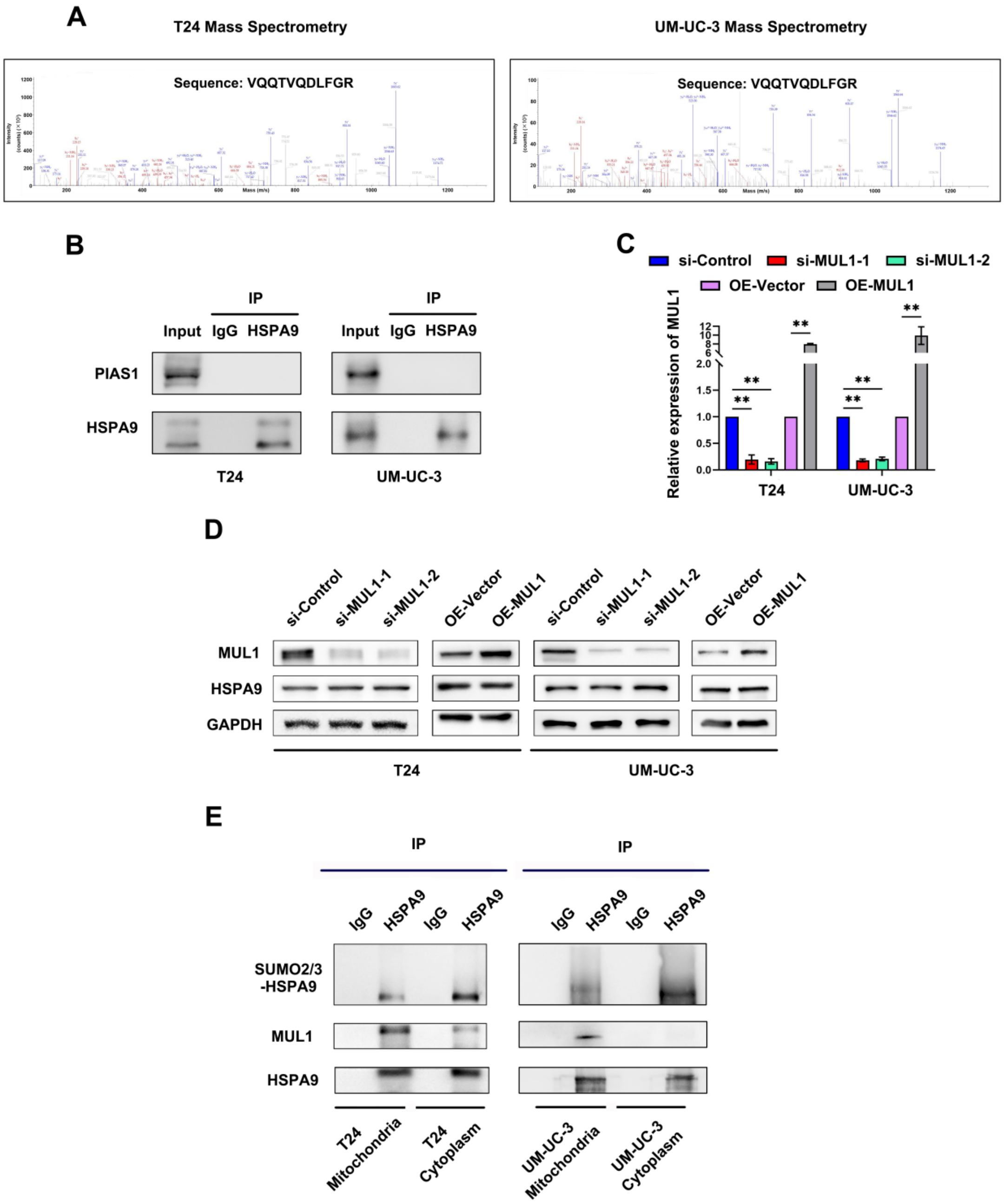

Fig. S1: MUL1 interacted with HSPA9. (A). Anti-MUL1 IP coupled with MS analysis was performed in T24 and UM-UC-3 cell lysates. The secondary spectrum diagrams of HSPA9 were shown. (B). Anti-HSPA9 IP was performed in T24 and UM-UC-3 cell lysates. PIAS1 level were detected via western blotting. Input served as a positive control and IgG served as a negative control. (C-D). Knockdown and stable

overexpression efficiency of MUL1 detected by qPCR (C) and WB (D) in MUL1-silencing or MUL1-overexpressing cells. HSPA9 and MUL1 levels was detected in MUL1-silencing or MUL1-overexpressing cells. (E). Mitochondrial and cytosolic proteins in T24 and UM-UC-3 cells were separated. Anti-HSPA9 IP was performed in mitochondrial and cytosolic protein lysates. SUMO2/3, MUL1 and HSPA9 levels were detected via western blotting. IgG served as a negative control. One-way analysis of variance (ANOVA) or two-tailed t tests were used to assess statistical significance. The standard deviations of three independent experiments are represented by error bars. *, $P < 0.05$; **, $P < 0.01$.

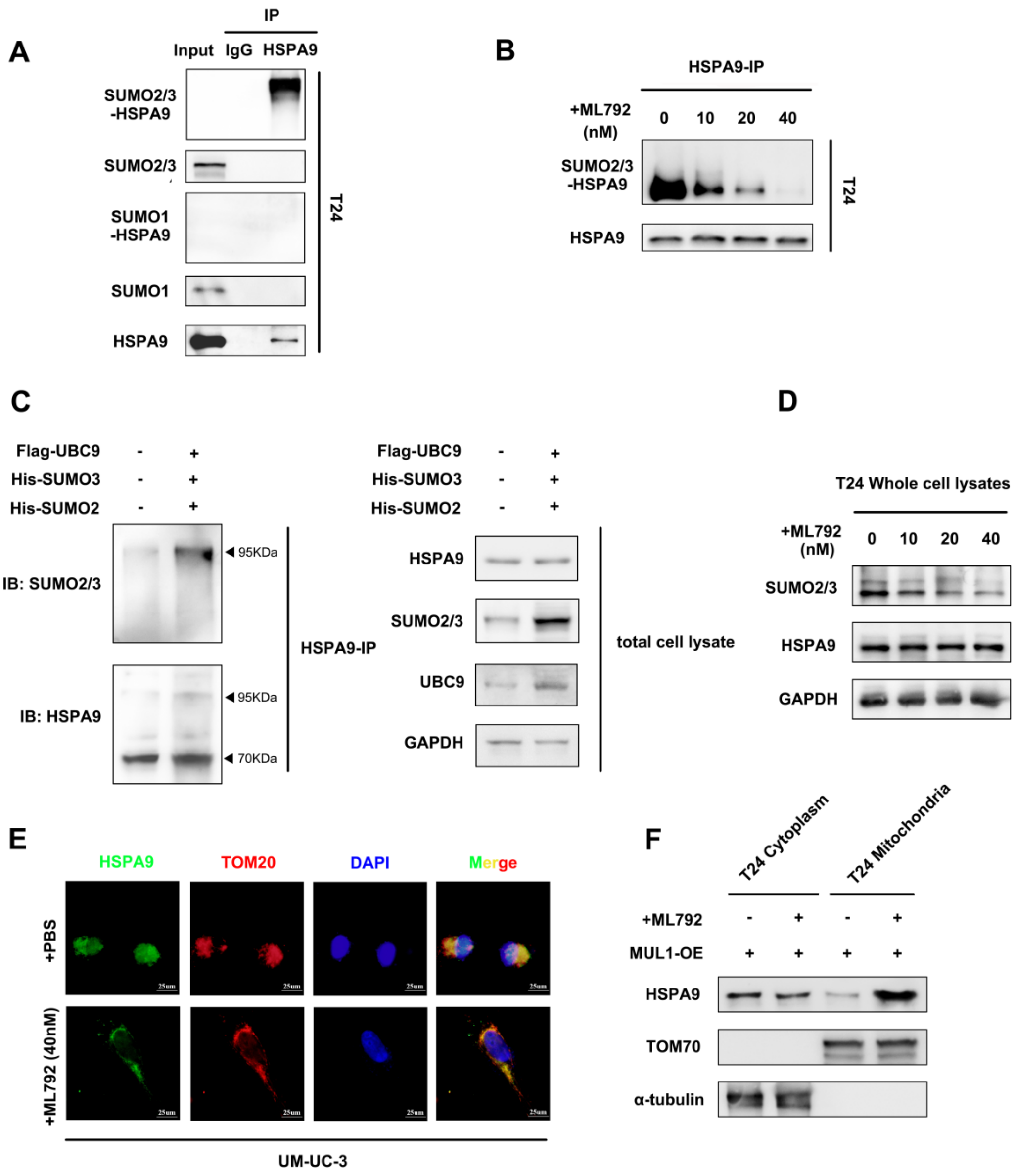

Fig. S2: MUL1 mediated the translocation of HSPA9 through SUMOylation. (A). Anti-HSPA9 IP was performed in T24 cell lysates. SUMO1 and SUMO2/3 levels were detected via western blotting. Input served as a positive control and IgG served as a negative control. (B). T24 cells were treated with ML792 at the concentrations indicated in the images. Anti-HSPA9 IP was performed in cell lysates. SUMO2/3 level was detected via western blotting. (C). Flag-UBC9, His-SUMO2 and SUMO3 was transient transfected into 293T cells. Anti-HSPA9 IP was performed to detect

SUMOylation level of endogenous HSPA9. SUMO2/3 and HSPA9 levels were detected. UBC9, SUMO2/3, GAPDH and HSPA9 levels in total cell lysates were shown. (D). T24 cells were treated with ML792 at the concentrations indicated in the images. Whole-cell lysates were prepared. HSPA9 and SUMO2/3 levels were detected via western blotting. (E). Representative IF images of HSPA9 localization in UM-UC-3 cells with the treatment of ML792 are shown. Green: HSPA9; red: TOM20; blue: nuclei (DAPI). Scale bars are shown in the right corner of the images. TOM20 was used as a mitochondrial tracer. (F). MUL1-overexpressing cells were treated with 20 nM ML792. HSPA9 levels in mitochondria and mitochondria-free cell fractions were detected via western blotting. TOM70 was used as a reference for mitochondrial proteins, and α-tubulin was used as a reference for cytosolic proteins.

Fig. S3: MUL1 catalyzed SUMOylation of HSPA9 at K612 site. (A). Overexpression efficiency of wild-type HSPA9 and HSPA9-K612R was detected by qPCR in OE-HSPA9 and OE-HSPA9-K612R cells. (B). HSPA9-K612R, HSPA9-K291R and HSPA9-K316R which were all tagged by GFP were overexpressed to generate corresponding OE-HSPA9-K612R, OE-HSPA9-K291R and OE-HSPA9-K316R cells. GFP-IP was separately performed in OE-HSPA9-K612R, OE-HSPA9-K291R and OE-HSPA9-K316R cell lysates. SUMO2/3 level was detected via western blotting.

(C). Anti-GFP IP was performed in GEP-Vector, OE-HSPA9 and OE-HSPA9-K612R cell lysates. MUL1 level was detected via western blotting. (D). Flag-UBC9, His-SUMO2 and SUMO3, GFP-HSPA9 or GFP-HSPA9-K612R was transient transfected into 293T cells. Anti-GFP IP was performed to detect SUMOylation level of exogenous HSPA9. SUMO2/3 and HSPA9 levels were detected. UBC9, SUMO2/3, GAPDH and HSPA9 levels in total cell lysates were shown. (E). Nuclear and mitochondria-free cytoplasmic faction of OE-HSPA9 and OE-HSPA9-K612R cells were separated. GFP level were detected via western blotting. Lamin B1 was used as a reference for nuclear proteins and α-tubulin was used as a reference for mitochondria-free cytoplasmic proteins. One-way analysis of variance (ANOVA) or two-tailed t tests were used to assess statistical significance. The standard deviations of three independent experiments are represented by error bars. *, $P < 0.05$; **, $P < 0.01$.

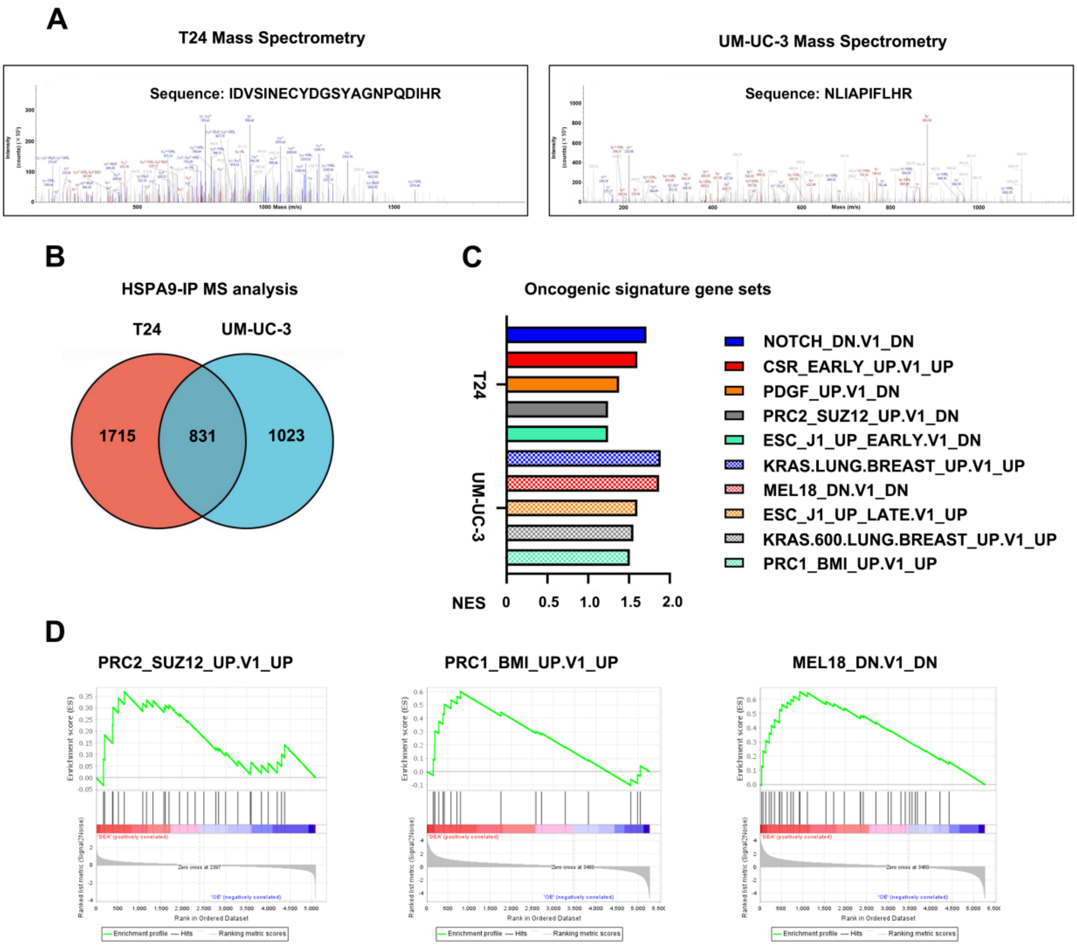

Fig. S4: Mutation of HSPA9 K612 site was relevant to PRC2 signaling pathway. (A). Anti-HSPA9 IP coupled with MS analysis was performed in T24 and UM-UC-3 wild type cell lysates. The secondary spectrum diagrams of SUZ12 were shown. (B). Identification of HSPA9-interacting proteins by MS analysis was shown in Venn diagrams. (C). Relative quantitative proteomics were performed to analysis differently expressed signaling pathways in OE-HSPA9 and OE-HSPA9-K612R cells. Histogram was plotted to show the top 10 differential signaling pathways which were analyses by GSEA and oncogene signature gene sets (C6). (D). GSEA revealed that mutation of HSPA9 K612 residue site was positive correlated to PRC associated pathways. The results were analyses and plotted by GSEA software.

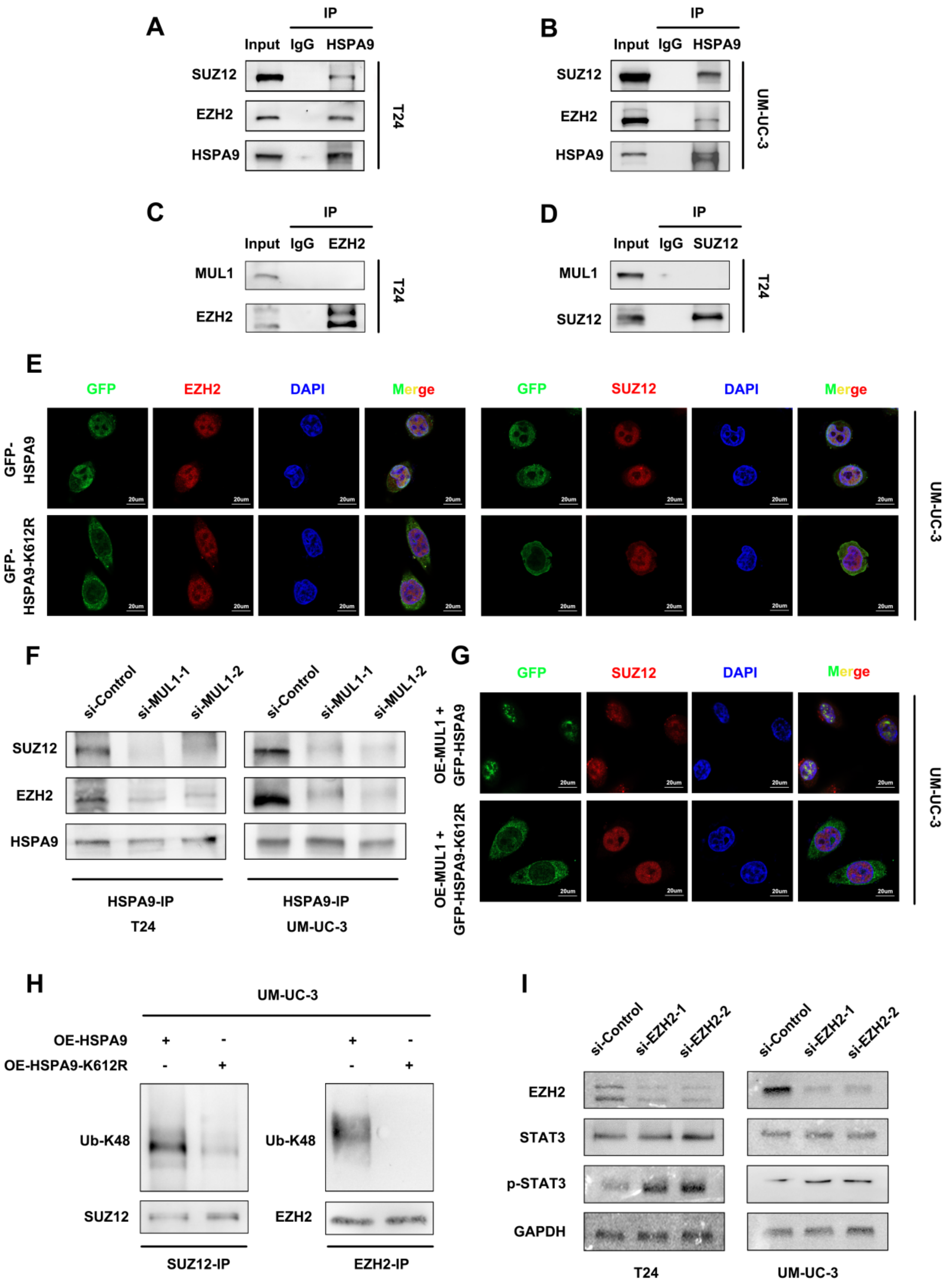

Fig. S5: SUMOylated HSPA9 interacted and induced the degradation of SUZ12 and EZH2. (A-B). Anti-HSPA9 IP was performed in T24 and UM-UC-3 cell lysates. SUZ12 and EZH2 levels were detected via western blotting. Input served as a positive

control and IgG served as a negative control. (C-D). Anti-SUZ12 IP and anti-EZH2 IP were performed in T24 cell lysates. MUL1 level was detected via western blotting. Input served as a positive control and IgG served as a negative control. (E). Representative IF images of GFP-SUZ12 and GFP-EZH2 colocalization in UM-UC-3 OE-HSPA9 and OE-HSPA9-K612R cells are shown. Both wild-type HSPA9 and HSPA9-K612R were tagged with GFP. Green: GFP; red: SUZ12/EZH2; blue: nuclei (DAPI). Scale bars are shown in the right corner of the images. (F). Anti-HSPA9 IP was performed in MUL1-silencing cell lysates. EZH2 and SUZ12 were detected via western blotting. Input served as a positive control and IgG served as a negative control. (G). Stable overexpression of vector or exogenous MUL1 was performed in UM-UC-3 OE-HSPA9 and OE-HSPA9-K612R cells. Both wild-type HSPA9 and HSPA9-K612R were tagged with GFP. Representative IF images of GFP and SUZ12 colocalization are shown. Green: GFP; red: SUZ12; blue: nuclei (DAPI). Scale bars are shown in the right corner of the images. (H). Anti-EZH2 and Anti-SUZ12 IP were performed in UM-UC-3 OE-HSPA9 and OE-HSPA9-K612R cell lysates. Ub-K48 level was detected via western blotting. (I). EZH2 was silenced in T24 and UM-UC-3 cells. STAT3 and p-STAT3 levels were detected via western blotting.

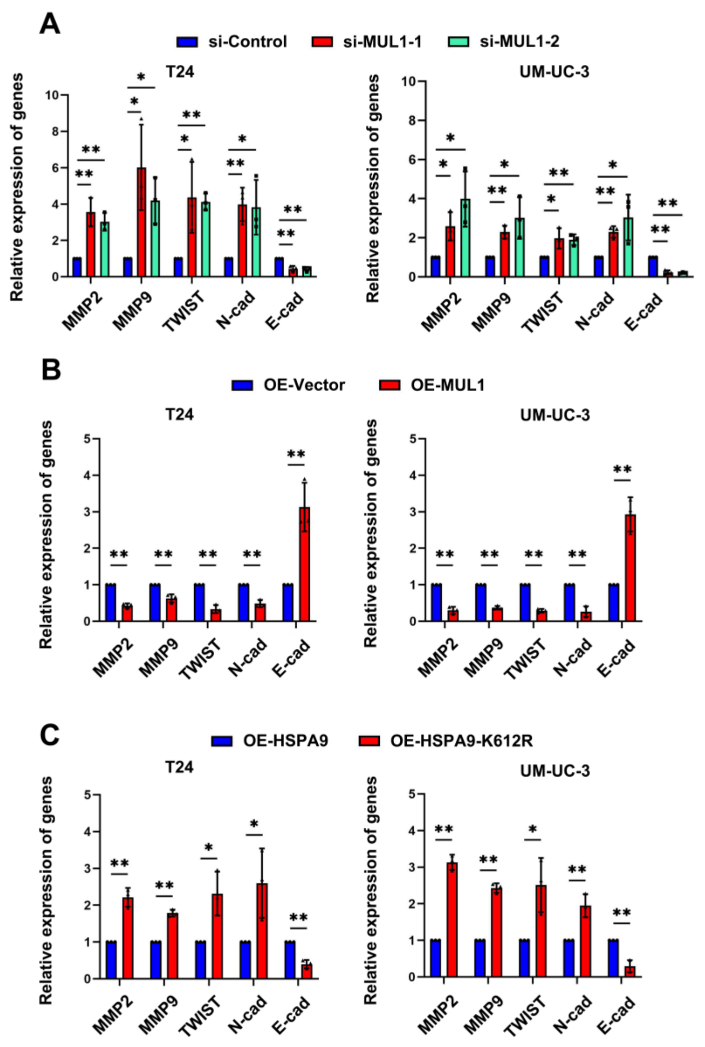

Fig. S6: SUMOylated HSPA9 and MUL1 inhibited STAT3 downstream metastasis-related genes. (A). MMP2, MMP9, TWIST, E-cad and N-cad levels were detected by qPCR in MUL1-knockdown cells. (B). MMP2, MMP9, TWIST, E-cad and N-cad levels were detected by qPCR in MUL1-overexpression cells. (C). MMP2, MMP9, TWIST, E-cad and N-cad levels were detected by qPCR in OE-HSPA9 and OE-HSPA9-K612R cells. One-way analysis of variance (ANOVA) or two-tailed t tests were used to assess statistical significance. The standard deviations of three independent experiments are represented by error bars. *, $P < 0.05$; **, $P < 0.01$.

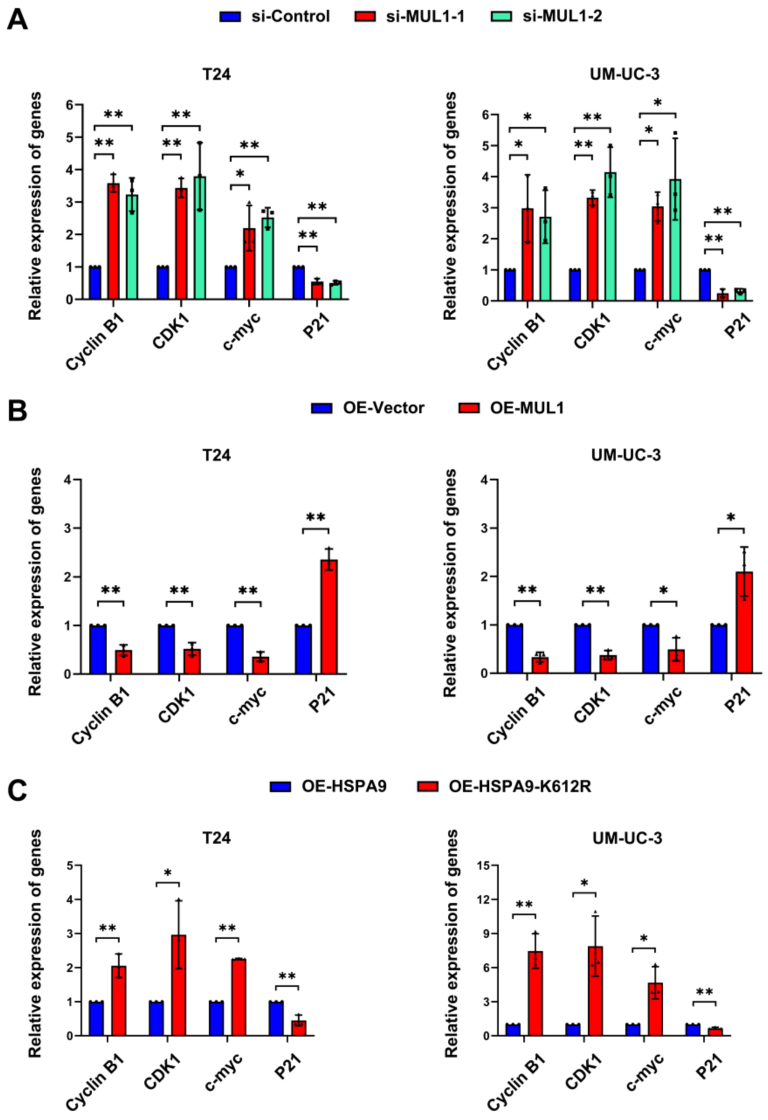

Fig. S7: SUMOylated HSPA9 and MUL1 inhibited STAT3 downstream proliferation-related genes. (A). Cyclin B1, CDK1, c-myc and P21 levels were detected by qPCR in MUL1-knockdown cells. (B). Cyclin B1, CDK1, c-myc and P21 levels were detected by qPCR in MUL1-overexpression cells. (C). Cyclin B1, CDK1, c-myc and P21 levels were detected by qPCR in OE-HSPA9 and OE-HSPA9-K612R cells. One-way analysis of variance (ANOVA) or two-tailed t tests were used to assess statistical significance. The standard deviations of three independent experiments are represented by error bars. *, $P < 0.05$; **, $P < 0.01$.

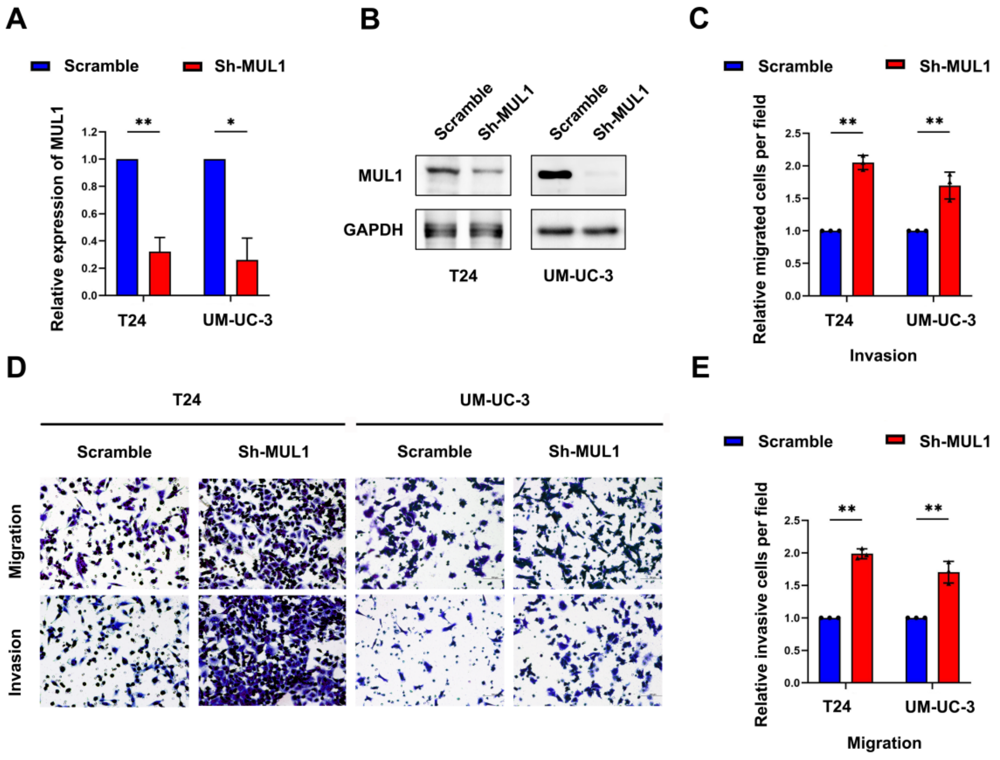

Fig. S8. Stable knockdown of MUL1 promoted migration and invasion of BCa cells. (A-B). Stable knockdown efficiency of MUL1 detected by qPCR (A) and WB (B) in MUL1 stable knockdown cells. (C-E). Representative images of migration and invasion assays of T24 and UM-UC-3 cells with MUL1 stable knockdown are shown in (D). The number of migratory and invasive cells in migration (C) and invasion (E) assays was measured and is shown in the histogram. One-way analysis of variance (ANOVA) or two-tailed t tests were used to assess statistical significance. The standard deviations of three independent experiments are represented by error bars. *, $P < 0.05$; **, $P < 0.01$.

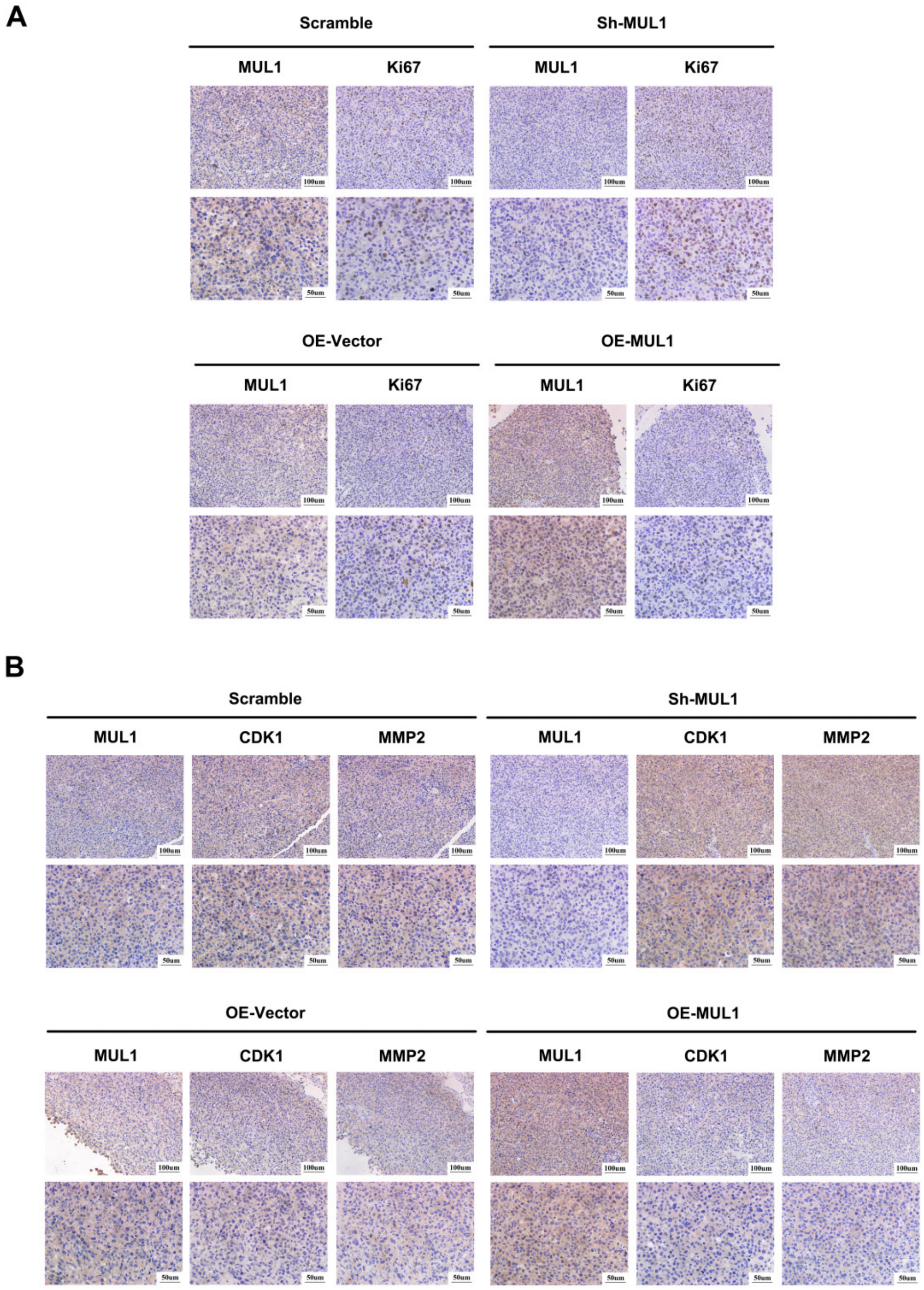

Fig. S9. MUL1 inhibited bladder cancer proliferation and LN metastasis *in vivo*. (A). IHC staining assays were used to detect Ki67 level in footpad tumors among different groups. (B). MUL1, CDK1 and MMP2 expression were separately measured by anti-MUL1, anti-CDK1 and anti-MMP2 IHC. One-way analysis of variance (ANOVA) or two-tailed t tests were used to assess statistical significance.

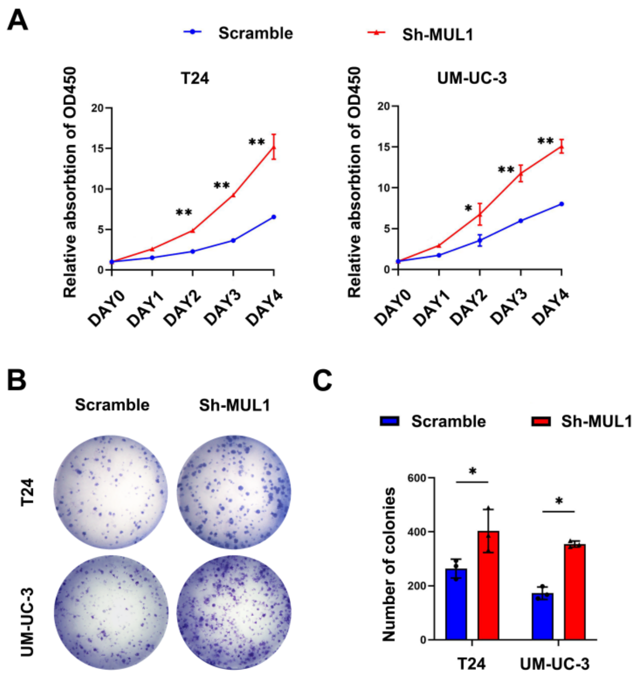

Fig. S10. Stable knockdown of MUL1 promoted proliferation of BCa cells. (A). Viability curves of T24 and UM-UC-3 cells with MUL1 stable knockdown were evaluated by CCK8 assays. (B-C). Representative images of colony formation assays of T24 and UM-UC-3 cells with MUL1 stable knockdown are shown in (B). The number of colonies were measured and is shown in histogram (C). One-way analysis of variance (ANOVA) or two-tailed t tests were used to assess statistical significance. The standard deviations of three independent experiments are represented by error bars. *, $P < 0.05$; **, $P < 0.01$.

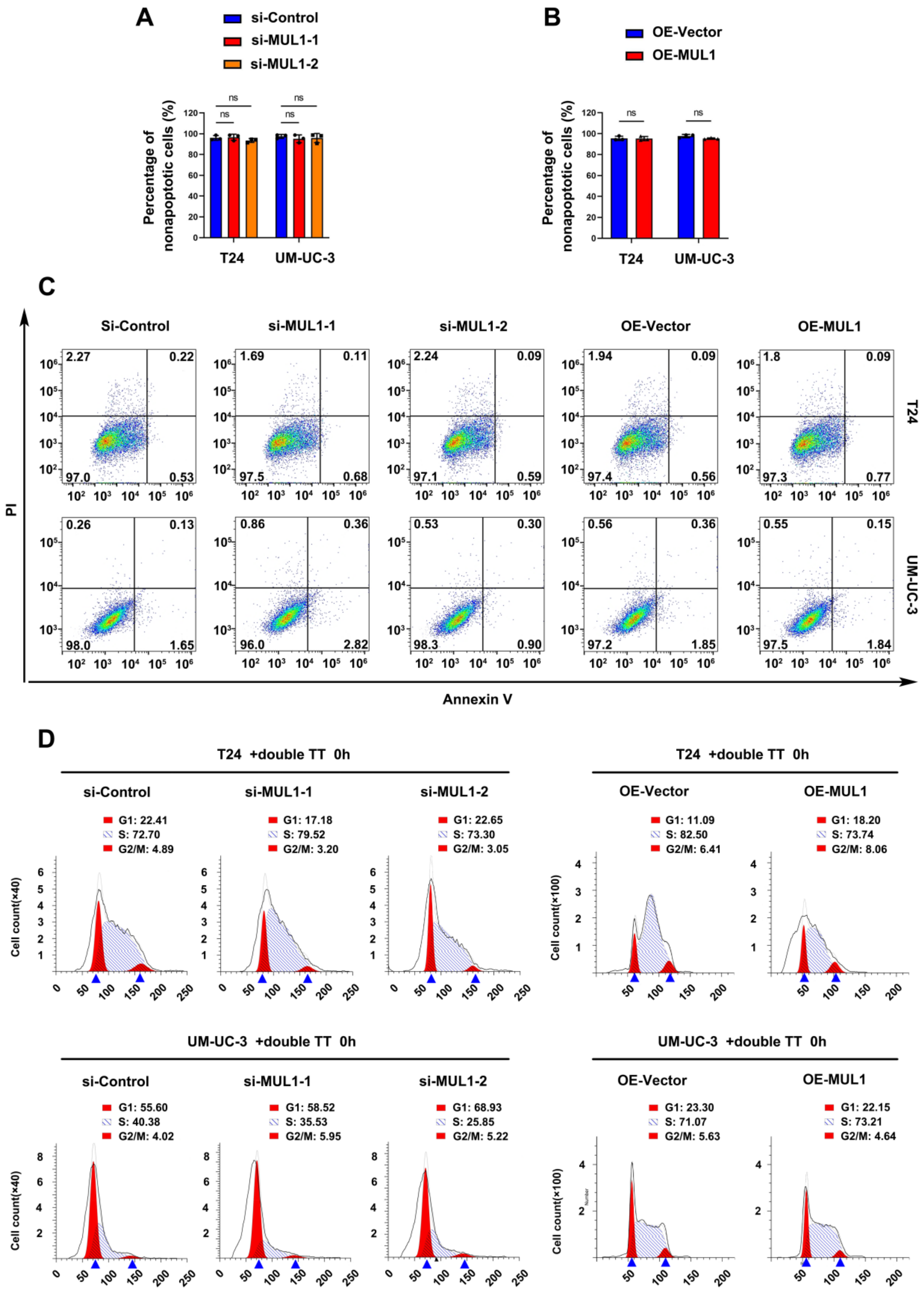

Fig. S11: MUL1 mediated G2/M phase arrest in BCa. (A-C). Apoptosis assays were performed in MUL1-silencing or -overexpressing cells. Statistics of percentages of nonapoptotic cells were shown in histogram (A, B). Representative images of

apoptosis assays are shown in (C). The X axis represents Annexin V channels and the Y axis represents PE-A channels in representative images. (D). Representative images of UM-UC-3 cell cycle synchronization assays. UM-UC-3 MUL1-silencing or -overexpressing cells were blocked with double thymidine. Cells were released for 6 or 7.5 hours after double thymidine blocks. Cells before and after release were then collected to perform cell cycle assays. The X axis represents PE-A channels, and the Y axis represents cell counts. One-way analysis of variance (ANOVA) or two-tailed t tests were used to assess statistical significance. The standard deviations of three independent experiments are represented by error bars. *, $P < 0.05$; **, $P < 0.01$.

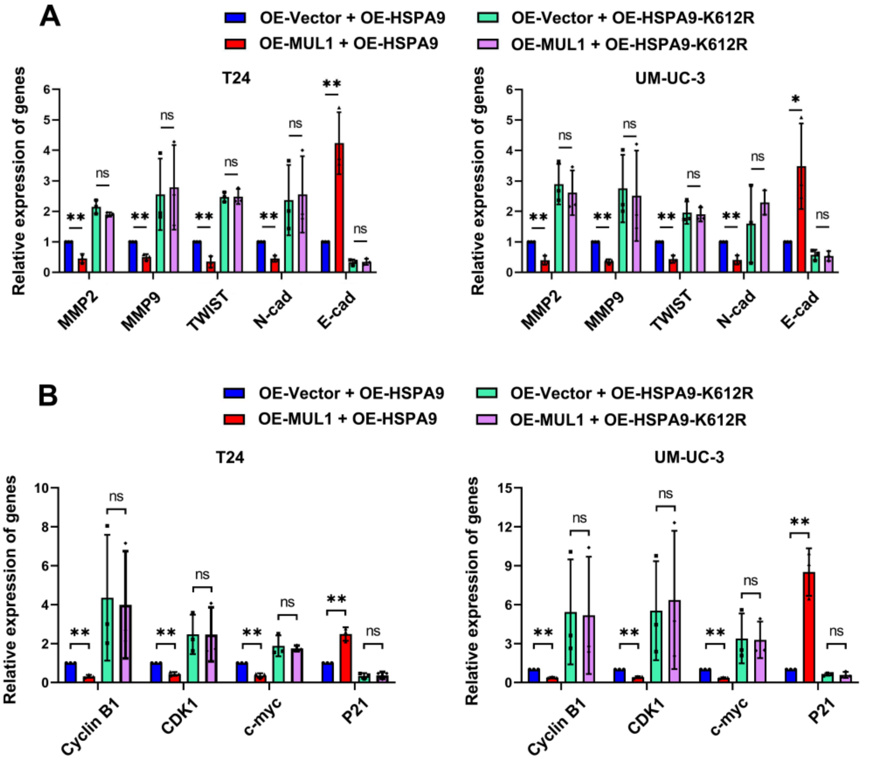

Fig. S12: Mutation of HSPA9 K612 site reversed MUL1-induced inhibition of STAT3 downstream metastasis-related genes and proliferation-related genes. (A). MMP2, MMP9, TWIST, E-cad and N-cad levels in OE-HSPA9 and OE-HSPA9-K612R cells overexpressing MUL1 or vector were detected via qPCR. (B). Cyclin B1, CDK1, c-myc and P21 levels in OE-HSPA9 and OE-HSPA9-K612R cells overexpressing MUL1 or vector were detected via qPCR. One-way analysis of variance (ANOVA) or two-tailed t tests were used to assess statistical significance. The standard deviations of three independent experiments are represented by error bars. *, $P < 0.05$; **, $P < 0.01$.

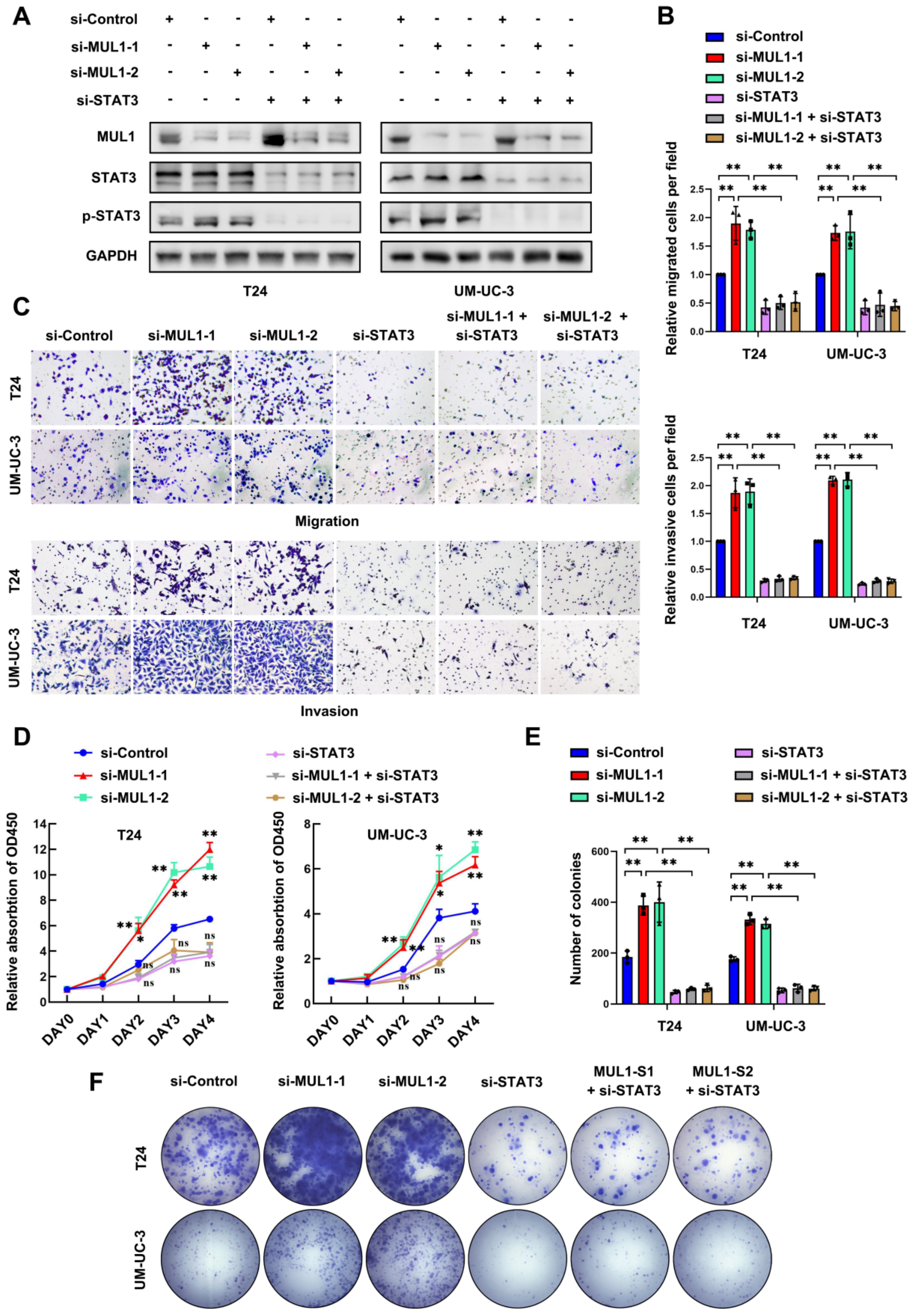

Fig. S13: Inhibition of STAT3 signaling reversed MUL1-induced tumor suppressive effects in BCa. (A). STAT3 was silenced in MUL1-silencing cells. The knockdown efficiency of STAT3 and p-STAT3 level were detected via western blotting. (B-C).

STAT3 was silenced in MUL1-silencing cells. Representative images of migration and invasion assays are shown in (C). The number of migratory and invasive cells in migration and invasion assays was measured and is shown in histogram (B). (D) STAT3 was silenced in MUL1-silencing cells. CCK8 assays were performed and viability curves were constructed using relative multiples of the relative absorption at 450 nm. (E-F) Representative images of colony formation assays which explored STAT3 function in MUL1-silencing cells are shown in (F). The number of colonies was measured and is shown in histogram (E). One-way analysis of variance (ANOVA) or two-tailed t tests were used to assess statistical significance. The standard deviations of three independent experiments are represented by error bars. *, $P < 0.05$; **, $P < 0.01$.

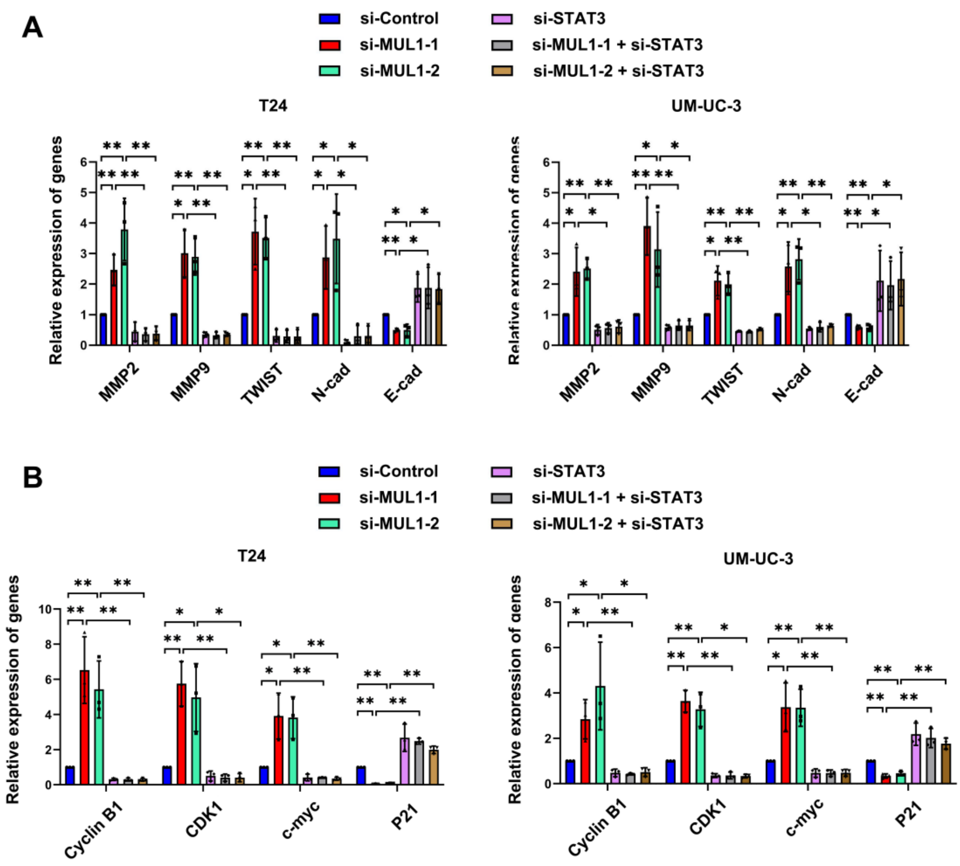

Fig. S14: Inhibition of STAT3 reversed MUL1-induced inhibition of STAT3 downstream metastasis-related genes and proliferation-related genes. (A). STAT3 was silenced in MUL1-silencing cells. MMP2, MMP9, TWIST, E-cad and N-cad levels in each group were detected via qPCR. (B). STAT3 was silenced in MUL1-silencing cells. Cyclin B1, CDK1, c-myc and P21 levels in each group were detected via qPCR. One-way analysis of variance (ANOVA) or two-tailed t tests were used to assess statistical significance. The standard deviations of three independent experiments are represented by error bars. *, $P < 0.05$; **, $P < 0.01$.

Supplementary Table S1. Univariate and multivariate analysis of factors associated with overall survival in cohort 1

Variable	Univariate analysis			Multivariate analysis		
	**HR**	**95% CI**	***P***	**HR**[2]	**95% CI**	***P***
Gender (Male / Female)	0.832	0.162-4.264	0.825		NA	
Age, years (<65 / ≥ 65)	4.634	1.029-20.867	**0.046**	10.009	1.051-95.350	**0.043**
Tumor Stage (Ta–T1 / T2–T4)	9.843	1.028-94.275	**0.047**	7.995	1.051-21.003	**0.045**
Nodal metastasis (N0 / N1–N2)	8.348	1.609-43.326	**0.012**	4.699	1.615-39.587	**0.011**
MUL1 (low / high)	0.174	0.033-0.924	**0.040**	0.171	0.032-0.907	**0.038**

Variables associated with survival by univariate analyses were adopted as covariates in multivariate analyses. Significant *P*-values are shown in bold font. HR > 1, risk for death increased; HR < 1, risk for death reduced.

Supplementary Table S2. Univariate and multivariate analysis of factors associated with progression free survival in cohort 1

Variable	Univariate analysis			Multivariate analysis		
	**HR**	**95% CI**	***P***	**HR**[2]	**95% CI**	***P***
Gender (Male / Female)	1.028	0.187-5.659	0.975		NA	
Age, years (<65 / ≥ 65)	1.326	0.383-4.589	**0.656**		NA	
Tumor Stage (Ta–T1 / T2–T4)	4.489	1.007-20.008	**0.049**	4.529	1.026-19.986	**0.046**
Nodal metastasis (N0 / N1–N2)	8.663	1.840-40.783	**0.006**	8.357	1.884-37.073	**0.005**
MUL1 (low / high)	0.196	0.051-0.755	**0.018**	0.200	0.053-0.757	**0.018**

Variables associated with survival by univariate analyses were adopted as covariates in multivariate analyses. Significant *P*-values are shown in bold font. HR > 1, risk for death increased; HR < 1, risk for death reduced.

Supplemental Table S3: Antibody used in this study

Application	Protein	Manufacturer	Cat number	Dilution for usage
Western blotting	MUL1	Proteintech	16133-1-AP	1:500
Western blotting	HSPA9	Proteintech	14887-1-AP	1:1000
Wsetern blotting	EZH2	Proteintech	21800-1-AP	1:1000
Western blotting	GFP	Proteintech	50430-2-AP	1:1000
Western blotting	SUZ12	Proteintech	20366-1-AP	1:1000
Western blotting	P-STAT3	Abconal	AP0070	1:1000
Western blotting	STAT3	Proteintech	10253-2-AP	1:2000
Western blotting	α-tubulin	Abconal	AC007	1:2000
Western blotting	TOM70	Proteintech	14528-1-AP	1:1000
Western blotting	GAPDH	Proteintech	60004-1-lg	1:2000
Western blotting	Lamin B1	Proteintech	12987-1-AP	1:500
Western blotting	Ub-K48	Abconal	A18163	1:1000
Western blotting	SUMO1	Proteintech	10329-1-AP	1:1000
Western blotting	SUMO2/3	Proteintech	11251-1-AP	1:1000
Western blotting	PIAS1	Proteintech	23395-1-AP	1:1000
Western blotting	UBC9	Proteintech	10070-1-AP	1:1000
Immunohistochemistry	CDK1	Proteintech	19532-1-AP	1:200
Immunohistochemistry	MMP2	ab86607	Abcam	1:200
Immunohistochemistry	MUL1	Proteintech	16133-1-AP	1:200
Immunohistochemistry	P-STAT3	Abconal	AP0070	1:200
Immunohistochemistry	EZH2	Abcam	#5246	1:400
Immunohistochemistry	SUZ12	Abconal	20366-1-AP	1:400
Immunoprecipitation	MUL1	Proteintech	16133-1-AP	4 μg/mL
Immunoprecipitation	HSPA9	Proteintech	14887-1-AP	4 μg/mL
Immunoprecipitation	SUZ12	Proteintech	20366-1-AP	4 μg/mL
Immunoprecipitation	EZH2	CST	#5246	4 μg/mL

Immunoprecipitation	GFP	Proteintech	50430-2-AP	4 μg/mL
immunofluorescence	MUL1	Abcam	ab155511	1:50
immunofluorescence	HSPA9	Proteintech	14887-1-AP	1:50
immunofluorescence	GFP	Proteintech	50430-2-AP	4 μg/mL
immunofluorescence	SUZ12	Abconal	A7786	1:50
immunofluorescence	EZH2	CST	#5246	1:50
immunofluorescence	TOM20	Proteintech	11802-1-AP	1:50

Supplemental Table S4: Primers used in this study

Primer Name	Sequence-Forward	Sequence-Reverse
MUL1	AAAGGCATCCAAGAGACCGA	ATGAAGAAGAGGGTGGCACA
HSPA9	GGATGTGCTGCTCCTTGATG	TCCACTTGCGTTTGACCATC
MMP2	ACCACAGCCAACTACGATGA	GCTCCTGAATGCCCTTGATG
MMP9	GCGTCTTCCCCTTCACTTTC	ATAGGGTACATGAGCGCCTC
TWIST	AGTCTTACGAGGAGCTGCAG	AGGAAGTCGATGTACCTGGC
N-cad	CTTGCCAGAAAACTCCAGGG	TGTGCCCTCAAATGAAACCG
E-cad	CGGACGATGATGTGAACACC	TTGCTGTTGTGCTTAACCCC
CCNB1(Cyclin B1)	TGAGGAAGAGCAAGCAGTCA	GCATCTTCTTGGGCACACAA
CDK1	ATGAAGTGTGGCCAGAAGTG	TCAGTGCCATTTTGCCAGAA
c-myc	GCACAAGAGTTCCGTAGCTG	GCACAAGAGTTCCGTAGCTG
P21	CCCAAGCTCTACCTTCCCAC	CTGAGAGTCTCCAGGTCCAC
GAPDH	GGATTTGGTCGTATTGGGCG	ATCGCCCCACTTGATTTTGG

Supplemental Table S5: Sequences of siRNAs and shRNAs in this study

Name	Sequence
**siRNA**	

Si-MUL1-1	GCCUUAUGCUGUUAUAGAATT
Si-MUL1-2	GGAAUGAUUGCUCAAAGAUTT
Si-STAT3	AACAUCUGCCUAGAUCGGCUATT
si-Control	UUCUCCGAACGUGUCACGUTT
**shRNA**	
MUL1-Sh	GCUUCAAGUCCUGCGUCUUTT
scramble	UUCUCCGAACGUGUCACGUTT
